# Deep learning driven colorectal polyp analysis: a review of detection, classification and segmentation methods

**DOI:** 10.3389/frai.2026.1786679

**Published:** 2026-06-03

**Authors:** Divya S, Sudha M

**Affiliations:** School of Computer Science Engineering and Information Systems, Vellore Institute of Technology, Vellore, India

**Keywords:** colorectal polyp analysis, computer aided diagnosis, deep learning, medical image analysis, polyp classification, polyp detection, polyp segmentation

## Abstract

Colorectal polyps are key determinants of colorectal cancer. Their accurate detection during colonoscopy has been a technically challenging work due to differences in shape size imaging conditions and texture. Emerging advances in Artificial Intelligence predominantly in deep learning have been making significant changes in the automatic detection and classification of polyps. This review presents a systematic and in-depth analysis of artificial intelligence-based methods for colorectal polyp detection classification and segmentation. Publicly available datasets are extensively reviewed along with data pre-processing and augmentation techniques that highlights low contrast noise and class imbalance. The review also investigates about the present state-of-the-art models for all three tasks. It is based on architecture designs performance trends and relative strengths. A thorough assessment has been made for the standard performance metrics used in existing literature for fair and consistent benchmarking. Finally existing gaps and future research paths have been discussed with an objective to fill the performance-translation gaps between experimental performance and clinical deployment. This review gives a structured reference for AI-based colorectal polyp analysis.

## 1 Introduction

Colorectal Cancer (CRC), better known as colon or bowel cancer, ranks the third most frequent type of cancer worldwide. It is emerging as the second major reason for cancer deaths (World Health Organization, 2023). From observations by the International Agency for Research on Cancer, it can be noted that the global burden of CRC remains considerable. It affects both developed countries and emerging nations (International Agency for Research on Cancer, n.d.). It has also been foreseen that the perceived global incidence of CRC will dramatically reach over 3.2 million cases every year by 2040 (Xi and Xu, 2021). A vast majority of patients develop CRC over a period of years due to the adenocarcinoma sequence. The benign polyps within the colon or rectum progress into malignancy. Early polyps, especially adenomas and sessile serrated polyps, are generally non-symptomatic, provided the opportunity for successful cancer prevention. Early identification and complete excision of polyps can prevent a vast majority of patients from developing colorectal cancer (Haggar and Boushey, 2009). Conversely, incomplete polyp resection or missed lesions may progressively grow in size, become dysplastic, invade the deeper layers of the colon, and may finally give rise to the formation of metastasis. Resulting in an advanced illness with a poorer prognosis, a higher cost of treatment, and a lower chance of survival (Morgan et al., 2023; (Hossain et al., 2022).

Colonoscopy is one of the important tools for colorectal cancer screening, offering both direct visualization and removal of precancerous lesions. However, its effectiveness in everyday clinical practice remains limited by several human performance factors (Gimeno-Garcia and Quintero, 2023; Keum and Giovannucci, 2019). These include rapid camera movement, poor lighting conditions, suboptimal bowel preparation, mucosal debris, and operator fatigue, which contributes to polyp miss rates, especially for subtle lesions such as flat and sessile serrated polyps (Mármol et al., 2017). These lesions have only a minimal appearance in the surrounding mucosa, display indistinct boundaries, and grow laterally rather than projecting into the lumen, these conditions make them very difficult to recognize even by experienced endoscopists. These missed lesions are one of the most common reasons for post-colonoscopy colorectal cancer. That still remains as a major and largely preventable clinical problem.

In recent times, deep learning techniques have widely been able to identify opportunities for application in the analysis of lesions during colonoscopy (Kather et al., 2019). These techniques include a convolutional neural network, Vision Transformers, hybrid CNN-Transformer models, generative models, and self-supervised learning methods that have proven effective in the automatic detection, segmentation, and classification of colon polyps (Tharwat et al., 2022). Research indicates that models based on CNNs can surpass traditional image processing methods in identifying small or subtle lesions. Recent research has concentrated on transformer-based contextual modelling, integrating features at multiple scales, and developing end-to-end multi-task learning frameworks. These advancements enable a single system to support real-time detection, accurate segmentation, and lesion characterization (Shin et al., 2018; Wang et al., 2020).

**Figure 1:**
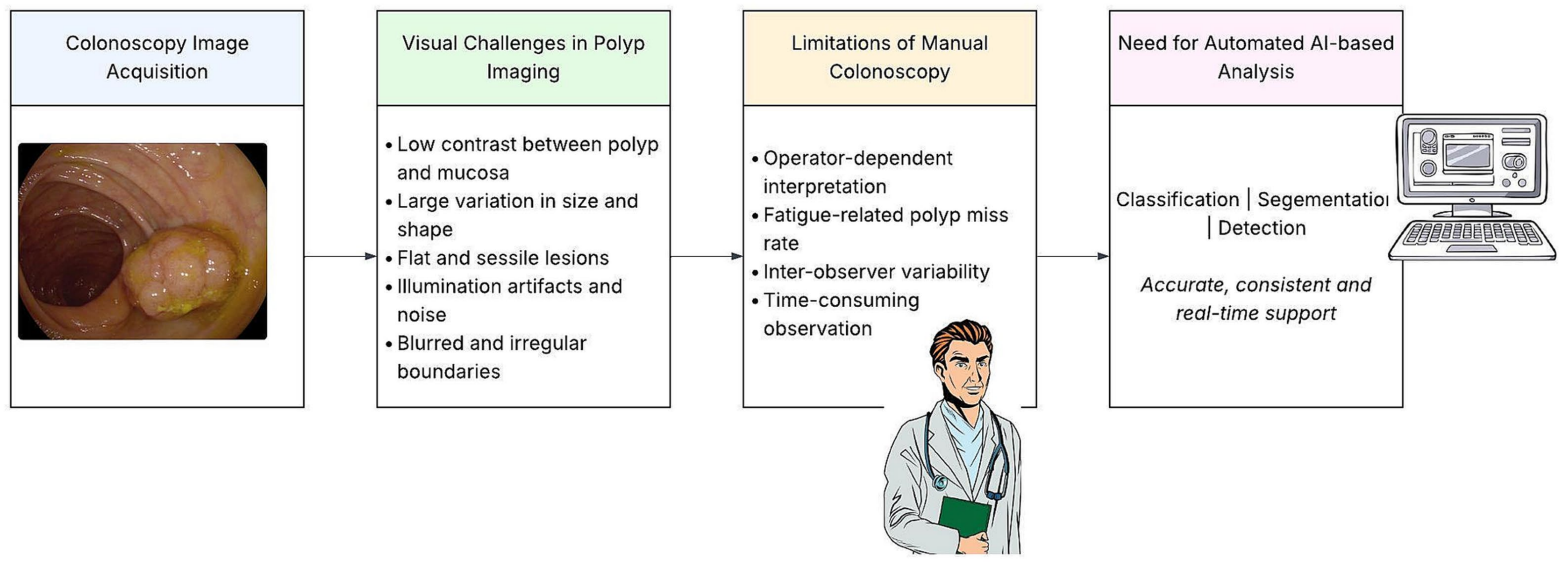
Clinical challenges and motivation for automated colorectal polyp analysis.

Despite the progress that has been made, the current state of research is quite fragmented. Detection, segmentation, and classification tend to be investigated individually, whereas they represent sequential tasks within one and the same clinical procedure. Furthermore, current methods are trained on datasets that vary in terms of annotation quality, preprocessing strategies, and used metrics of evaluation. More importantly, most research works tend to be conducted on small local databases that fail to consider the typical variations encountered in practical colonoscopy (Nisha et al., 2022; Lee et al., 2020). Figure 1 explains the challenges in polyp imaging, limitations of manual colonoscopy and need for an automated AI-based analysis.

This review presents an overall synthesis of recent deep learning works in colonoscopy, focusing on model architectures, datasets, pre-processing workflows, and evaluation protocols. A critical appraisal of the most recent emerging approaches, including hybrid CNN-Transformer models, GAN-based synthetic data generation, and self-supervised contrastive learning, is conducted to assess their impact on robustness, generalization, and clinical relevance. The paper further discusses the need for integrated, real-time, uncertainty-aware Holistic Colonoscopy Intelligence systems that can deliver stable, interpretable, and clinically relevant assistance during the entire colonoscopy procedure.

This review aims to provide a cohesive, clinically-aligned presentation on the latest and future of deep learning in colonoscopy. Informs the research and industry communities on how to utilize artificial intelligence for early detection of polyp growth. Also provide details on prevent missed lesions, prevent the progression to colorectal cancer. That ultimately advance patient care through these technologies.

To facilitate a cohesive and inclusive integration of the literature for a meaningful synthesis, this review will follow a structured format to address eight research questions that encompass the key parameters required for the development of clinically valid AI solutions for colonoscopy tasks. The questions will address the limitations and potential of existing techniques to point out the gaps in the literature and unfold areas for future development to reach integrated and clinically viable solutions.

1. How are the current state-of-the-art models and architectures for Deep Learning applied for the analysis of colon polyps different from each other?
2. How do various DL architectures handle a variety of publicly available and medical datasets under different imaging conditions?
3. Which preprocessing and data augmentation methods are used for improving robustness and generalizability and for making a model ready for clinical use?
4. What are the major evaluation criteria used in practice, and how do these indicate the practical applications of the model?
5. How successful are unified or multi-task models in comparison with task-specific models?
6. How key challenges like small polyp detection, temporal consistency, real-time performance and uncertainty estimation restrict the implementation in clinical practice?
7. What are the major issues, limitations, and research gaps in the existing literature with respect to the quality of datasets, generalization performance, and computational cost.?
8. How is explainability and interpretability incorporated into DL models for easier clinical acceptance?
9. What are the upcoming potential research areas that are most crucial for improving clinical deployment and real-world acquisition?

**Figure 2:**
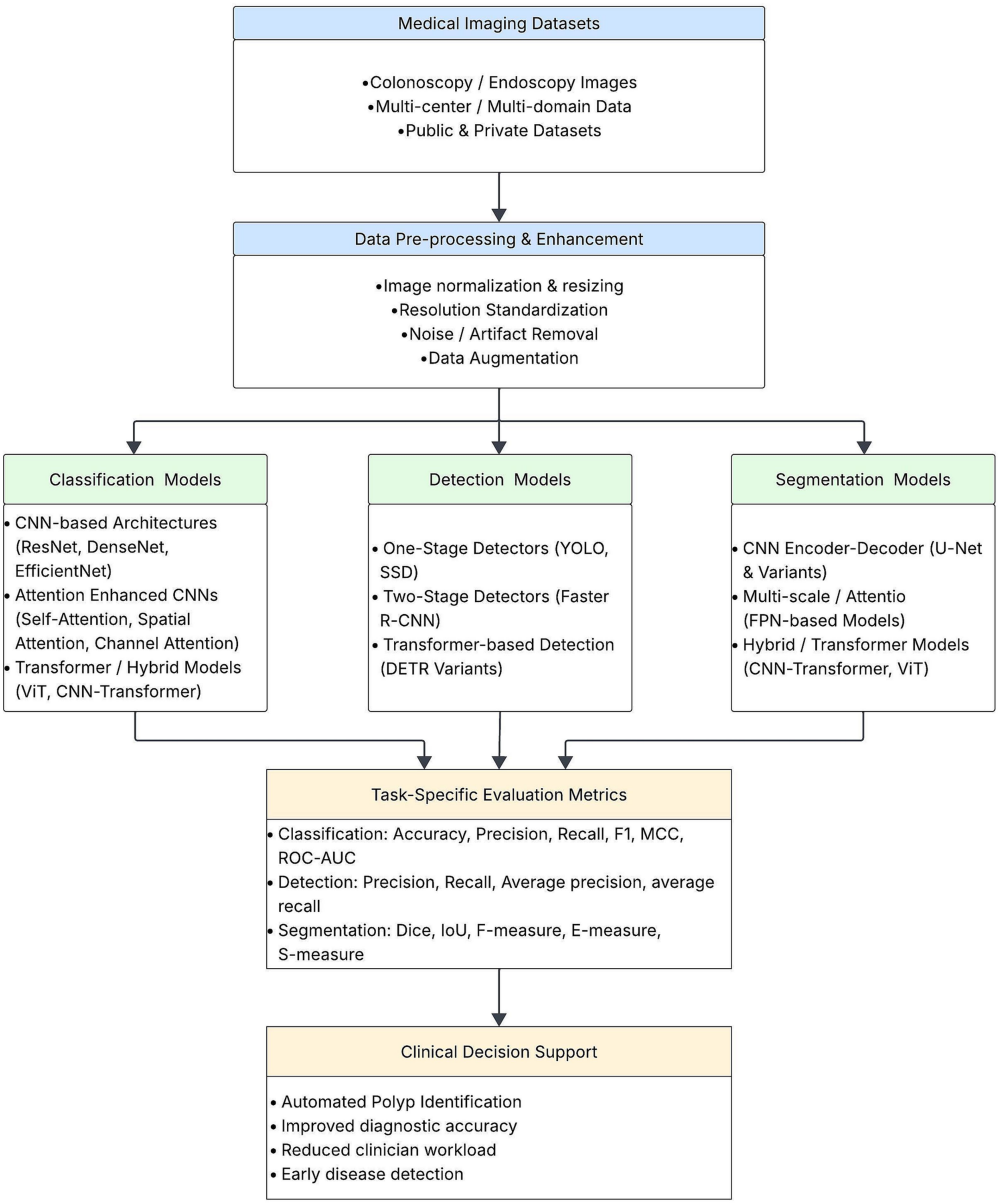
Deep learning framework for medical imaging.

Figure 2 gives a overview of deep learning framework for medical imaging.

## 2 Methodology

### 2.1 Eligibility criteria

The criteria of eligibility have been formalized to maintain methodological accuracy and relevance. The studies must be written in English and peer-reviewed. The articles must be published within the previous decade. The relevance of the articles includes research on patient-derived colonoscopy images or video that is used by deep learning-based models in detection, segmentation and classification of colorectal polyps. The articles must have quantitative performance comparisons to verify their accuracy. The studies included all ages of patients to maintain relevance in a practical medical environment. The eligibility criteria are summarized in Table 1.

**Table 1:** Eligibility criteria.

Criteria	Definitions
Paper language	English
Publication period	2015–2025
Study type	Peer-reviewed journal articles and full conference papers.
Participants age	No age-related restrictions
Application area	Deep learning-based colonoscopy image and videos analysis for colon polyp detection, segmentation and classification
Domain	Medical image analysis Deep learning Artificial intelligence Computer aided diagnosis
Imaging modality	Colonoscopy (white-light imaging, narrow-band imaging)
Data types	Images or video sequences
Learning paradigms	Supervised learning Self-supervised learning Semi-supervised learning Weakly supervised learning

### 2.2 Inclusion and exclusion criteria

To ensure the selection of relevant and scientifically valid studies, a structured approach to the formulation of inclusion and exclusion criteria was developed before commencing the review process. The structured approach was developed to ensure a consistent approach to screening the literature for relevance to this review and to ensure alignment with the aims of this review. The structured approach assisted in the selection of studies for this review and helped ensure a level of comparison and science validity among the body of work selected for this investigation. A summary of the inclusion and exclusion criteria is presented in Table 2.

**Table 2:** Inclusion exclusion criteria.

Criteria	Inclusion	Exclusion
Study significance	Colorectal polyp detection, segmentation, classification using deep learning.	Studies unrelated to colorectal polyps.
Technological extent	Deep learning–based approaches including CNNs, vision transformers, hybrid and multi-task architectures	Traditional machine learning or rule-based methods.
Data type	Clinical colonoscopy images or video data may include public or institutional datasets	Synthetic, simulated, or non-clinical data without validation on real colonoscopy data
Imaging modality	*In vivo* colonoscopy (white-light imaging, narrow-band imaging)	CT colonography, histopathology-only, or non-endoscopic imaging modalities
Publication type	Peer-reviewed journal articles and full conference papers	Preprints, non–peer-reviewed works, editorials, book chapters, and workshop abstracts
Methodology	Empirical studies proposing or evaluating deep learning models with quantitative benchmarking	Studies lacking experimental evaluation or comparative performance analysis
Evaluation	Studies reporting standard quantitative performance metrics	Studies without clear performance reporting
Clinical relevance	Methods addressing real-time or clinically applicable workflows	Purely theoretical studies without clinical context.

### 2.3 Search strategy

An extensive literature assessment was undertaken to discover appropriate research on colon polyp detection, classification and segmentation using various scientific journal databases that includes IEEE, Web of Science, Google Scholar, Scopus, ScienceDirect, ACM Digital Library, PubMed, SpringerLink, Wiley Online Library. The search strategy comprises combination of keywords “colon,” “polyps,” “colorectal,” “colon cancer,” “adenoma,” “colonoscopy,” “detection,” “classification,” “segmentation,” “deep learning,” “CNN,” “transformers,” “attention mechanism,” “explainable AI,” “self-supervised,” “autonomous,” “gastrointestinal polyps,” “neoplasia,” “semi-supervised,” “images,” “colorectal lesions,” “localization,” “artificial intelligence,” “supervised,” “multi-class.” To uncover and gather journals associated to the ranges that were the centre of this review Boolean operators like AND, OR were used. The absolute collection of papers was concluded by their influence with colon polyp research, scientific rigour and significance.

### 2.4 Study quality assessment

**Figure 3:**
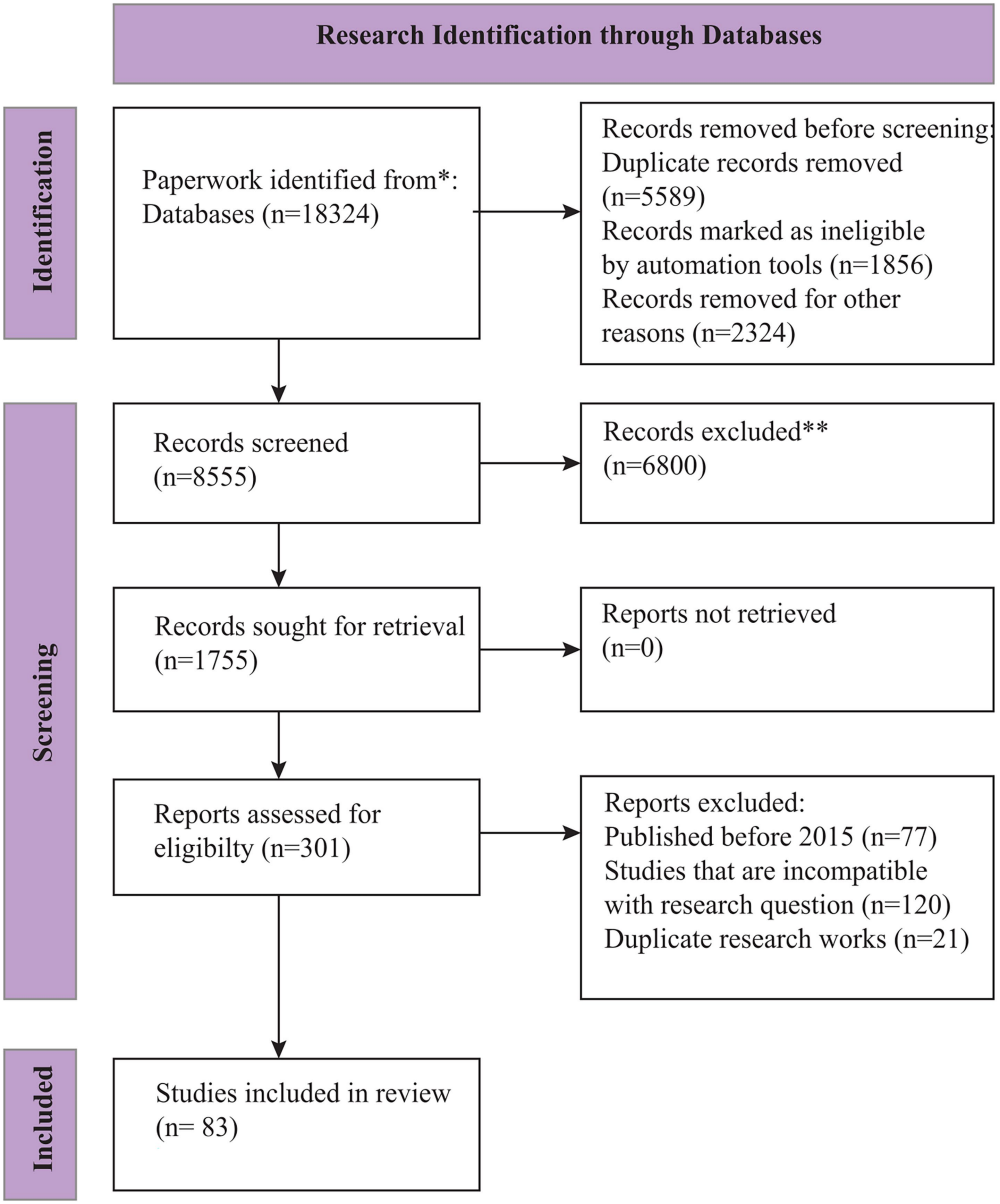
PRISMA diagram.

The PRISMA diagram, which summarizes the stages from primary research identification to the last inclusion of studies delivers a representation of the systematic review process. A total of 18,324 paperwork has been identified from the scientific databases. Some records with duplicates, marked as ineligible by automations tools were removed before screening. The record screened include 8,555. Further certain papers were excluded and finally 301 works were assessed for eligibility. 218 papers that are incompatible with research question and redundant research works were removed. Eventually 83 paper works were incorporated in the final review. Figure 3 is the PRISMA diagram.

### 2.5 Data extraction

To enable a structured and comparative analysis of the results, pertinent data points were carefully extracted from each respective study. These data points included information about the image data sets, including the nature of such sets, that is, public or private, with respect to size and image characteristics, and the methods of data preparation, that is, normalization, resizing, artifact removal, illumination change, and creation of virtual data. Details about the architecture of the models, that is, whether the models were of the CNN, Transformer, or combined category, the use of attention modules, the approach taken toward multi-task learning, and ensemble methods, were also noted. Details of the training methods, that is, the optimizer, loss function, number of samples, number of epochs, and regularization methods, were also taken. Furthermore, details of evaluation metrics and performance, that is, detection, segmentation, and classification, were also extracted, and details about the methods of explanation, that is, its relation to clinical explanation, were also extracted. Details of the respective advantages, limitations, and challenges were also extracted from each respective study.

## 3 Data sources and dataset characteristics

### 3.1 Colonoscopy datasets

Datasets play an integral part in the progress of medical image analysis. These datasets help carry out research work in a transparent and reproducible manner. They also help perform comparative analysis between studies. These datasets are shared openly for the benefit of the research community. These datasets may contain expert-annotated information and predefined data splits. In the context of colonoscopy and polyp analysis, the available dataset varied based on the size, shape, texture, and image parameters of the polyps. They also help perform reliable learning and testing on detection and segmentation tasks. The following describes the most commonly seen datasets and their salient features.

#### 3.1.1 Kvasir

Kvasir is a GI endoscopy dataset which contain images acquired from Vestre Viken Health Trust in Norway. All the images are quality assured and marked by senior endoscopists and representatives from the Cancer Registry of Norway. There eight different classes in the datasets which includes anatomical landmarks such as Z-line, pylorus, and cecum, along with clinical conditions such as esophagitis, polyps, and ulcerative colitis. It also includes images related to polyp removal techniques, such as dyed and lifted polyps or dyed resection margins. Images differ in resolution between 720 × 576 and 1920 × 1,072 (Pogorelov et al., 2017).

#### 3.1.2 Kvasir-SEG

Kvasir-SEG is an open-access dataset, intended for pixel-wise polyp segmentation in gastrointestinal endoscopy. It comprises 1,000 polyp images which were sourced from the Kvasir dataset. A ground-truth segmentation mask has been provided for each image. Size of each image vary between 332 × 487 and 1920 × 1,072 pixels. The masks are provided in 1-bit binary form. All the masks are manually created and verified by experienced gastroenterologists using the Labelbox annotation tool; bounding box coordinates are also specified in a JSON file. With Kvasir-SEG adding pixel-level segmentation to a dataset previously containing only frame-level labels, it allows robust benchmarking and development of advanced algorithms for the segmentation, detection, and localization of polyps (Jha et al., 2019).

#### 3.1.3 CVC-clinic-DB

This dataset includes 612 images obtained from 21 videos recorded from colonoscopies performed with white light video colonoscope. The videos were from 31 different polyps located within the 24 videos. Polyps are classified based on Paris classification as protruded (0-Is, 0-Ip) or flat (0-II). The images are in the size of 576 × 768 pixels (Bernal et al., 2015).

#### 3.1.4 CVC-Colon-DB

The dataset was created by Computer Vision Center and Computer Science Department of Universitat Autonoma de Barcelona, Spain. The images are taken from 15 short colonoscopy sequences from 15 cases. Random sample of 20 frames per sequence was acquired. Totally 300 images were selected with frame size 500 × 574 pixels. Labelling of the region of interest for each frame was provided (Bernal et al., 2012).

#### 3.1.5 ETIS-Larib polyp DB

This data is extracted from 34 colonoscopy video sequences. From these sequences 196 image frames are taken. These correspond to 44 different polyps across the 34 sequences. Each image is with a uniform resolution of 1,225 × 966 pixels. A binary mask was provided along with each image representing the polyp region. The annotation was done by expert endoscopists (Silva et al., 2014).

#### 3.1.6 BKAI-IGH NeoPolyp-small

This dataset was released by BKAI(HUST) in collaboration along with Institute of Gastroenterology and Hepatology (IGH), Vietnam. It is used for polyp segmentation, classification and neoplasm detection. It consists of 1,200 images which contains 1,000 WLI images and 200 FICE images. Background, non-neoplastic polyps and neoplastic polyps are the three classes founded in this dataset (Duc et al., 2022; An et al., 2022)

#### 3.1.7 Hyper-Kvasir

The Hyper-Kvasir dataset is a extensive gastrointestinal image and video dataset mainly used for medical image analysis. It contains 10,662 labelled images and 99,417 unlabeled images across 23 classes. A division of 1,000 polyp images includes segmentation masks and bounding boxes for segmentation related tasks. Additionally, it has 373 annotated videos labelled by medical experts. The dataset supports classification, detection, segmentation, and video-based research (Borgli et al., 2020).

#### 3.1.8 CVC-EndoSceneStill

CVC-EndoSceneStill is a comprehensive dataset, as it combines two previous datasets: CVC-ClinicDB and CVC-ColonDB. This dataset consists of 912 images, which are obtained from 44 different colonoscopy video sequences, taken from 36 different patients. Classes are related to different important parts of the images used in colonoscopy procedures. These classes are the polyp, the lumen of the colon, specular highlights due to light reflections, and a void class related to black borders of images. These additional classes make this dataset more interesting, as they allow the model to distinguish between real polyps and other possible image artifacts, such as glare or the lumen of the colon.

#### 3.1.9 CVC-300

CVC-300 is commonly used in polyp segmentation studies as a dataset for testing. It is also commonly known as a subset of the CVC-ColonDB dataset. CVC-300 comprises 300 images from 13 different polyp video sequences. CVC-300 is considered to be a relatively small dataset when compared to other large datasets available in recent times. The model is commonly trained using larger datasets like Kvasir-SEG. Then, CVC-300 is used to test the ability of the model to generalize to unknown data.

#### 3.1.10 CVC-T

CVC-T is a particular set of testing data from a larger set of data known as CVC-ClinicDB. The CVC-T set was established as a standardized test for a challenge in a 2015 MICCAI competition for polyp detection algorithms. In most studies, CVC-T refers to a set of 60 test images used for evaluating algorithms. The set is commonly used for evaluating the performance of a segmentation model.

#### 3.1.11 PICCOLO (polyp image collection for colonoscopy)

The images from this dataset are obtained from clinical colonoscopy videos. 3,433 white light and narrow banding images were obtained. It included 76 distinct lesions from 48 patients. For each lesion clinical metadata are given as number of polyps found, polyp identification, polyp size, Paris classification, NICE rating, Preliminary diagnosis, precise diagnosis, histological stratification. Annotation types include segmentation masks for polyps and bounding boxes for detection (Sánchez-Peralta et al., 2020).

#### 3.1.12 LDPolypVideo

This database contains 160 labelled colonoscopy videos. The labelled data is divided into 40,266 frames out of which 33,884 frames contain polyps and 6,382 is without polyps. The dataset also contains 103 unlabelled videos. In that unlabelled videos there are 42 polyp videos which are divided into 371, 400 polyp frames. Remaining 61 non-polyp videos are divided into 490,000 non-polyp frames (Ma et al., 2021).

#### 3.1.13 Sun

This dataset was by Showa University and Nagoya University (SUN), it is a colonoscopy video database. It contains 113 colonoscopy videos in which 100 are positive and 13 are negative. The videos are divided into frames which contains 49,136 polyp frames and 109,554 non-polyp frames. The annotations are bounding boxes and pathological findings that falls into six categories which are low-grade adenoma, high-grade adenoma, hyperplastic polyp, sessile serrated lesion, traditional serrated adenoma, invasive carcinoma (Misawa et al., 2021).

#### 3.1.14 Sun-SEG

It is a large-scale Video Polyp Segmentation dataset with frame-by-frame annotations. The source of the video is from Showa University and Nagoya database. From the original SUN database 1,106 videos are manually trimmed and collected. The videos can be divided into 378 positive and 728 negatives. The dataset consists of 158,690 video frames obtained from the clips. Five hierarchies of annotations are used 1. Visual attribute: Surgical Instruments (SI), Indefinable Boundaries (IB), Fast Motion (FM), Small Object (SO) and Occlusion (OC). 2. Object Mask: Each frame is provided with a pixel-wise masks. 3. Polyp boundary generation. 4. Scribble annotation. 5. Polygon annotation (Ji et al., 2022).

#### 3.1.15 Kvasir sessile

The Kvasir-Sessile dataset is a variant of the Kvasir-SEG dataset. The Kvasir-Sessile dataset is used for sessile polyps, which are flat or dome-shaped growths without a stalk or a clear boundary. The sessile growths are clinically significant because they can be pre-cancerous in nature and can be challenging to identify against the colon mucosal wall. The Kvasir-Sessile dataset is used as a challenging test case for a model’s ability to identify boundaries with low contrast. The Kvasir-Sessile dataset is used for enhancing the sensitivity of a computer-aided diagnosis system and reducing the number of missed cases in a clinical scenario.

#### 3.1.16 Kvasir-capsule

This dataset encompasses 47,238 labelled images and 117 videos. The dataset is partitioned into labelled videos, labelled videos and unlabeled videos. Labelled images are of 14 classes consist of bounding box annotations. Labelled videos consist of 43 annotated videos. Unlabeled videos are of 74 in number and they are of 25 h unannotated footage (Smedsrud et al., 2021).

#### 3.1.17 Gastrointestinal lesions in regular colonoscopy dataset

The Gastrointestinal Lesions in Regular Colonoscopy dataset is intended for the classification and characterization of lesions in the colon. This dataset is used to determine the histopathology of the lesions. The dataset contains 76 lesion sequences, which were obtained from 76 different types of polyps. Each lesion is captured using two different modalities: White Light and Narrow Band Imaging. The total number of video sequences is 152, as each lesion is recorded using two modalities. The dataset contains three types of lesions: adenoma, serrated adenoma, and hyperplastic. The main purpose of the GLRC dataset is to enable the development of virtual biopsy systems, which can be used to determine whether the lesion should be removed or left as it is.

#### 3.1.18 ASU-Mayo Clinic colonoscopy video database

This database is a collection of colonoscopy videos of 19,400 frames. 5,200 frames contain polyps and remaining 14,200 frames are without polyps. Each frame comes with binary ground truth image. The ground truth was created by medical experts. This dataset is not publicly available; potential users must request permission from the data provider at Arizona State University (Tajbakhsh et al., 2015).

#### 3.1.19 PolypGen

This dataset was from six different centers and collected from 300 patients. It contains 8,037 frames. The frames are divided into 3,762 positive samples and 4,275 negative samples. It provided high resolution segmentation masks and bounding box annotations. The images are of size from 384 × 288 to 1920 × 1,080. This dataset also included various size polyps (≤ 100 × 100 pixels), medium (>100 × 100 and ≤ 2000 × 200 pixels) and large (≥ 200 × 200 pixels) (Ali et al., 2023).

#### 3.1.20 EDD 2020

The dataset has been curated from 280 patient videos, collected across multiple organs and various clinical centers. There are 45,478 expert annotations in total in both single-image frames and video sequences. For the detection task, the training set contains 2,531 images with 31,069 bounding boxes, at the same time the segmentation task provides 643 frames with 7,511 binary masks. A small set of video sequences is also available, comprising 5 sequences for detection and 2 sequences for segmentation, along with some additional test sequences (Ali et al., 2020; Ali et al., 2021).

In addition to the commonly used datasets discussed above, there are some more datasets that have been proposed for the analysis of colorectal polyps. These datasets are PIBAdb (Colorectal Polyp Image Cohort), PolypSegm-ASH, the KUMC dataset, and POLAR DB. PIBAdb (Colorectal Polyp Image Cohort) dataset is contains images with variety of morphological changes, allowing the model to generalize better on the varied patient population. PolypSegm-ASH dataset is particularly designed for segmentation problems. It focuses on the boundaries and lighting changes commonly found during real-time colonoscopies. KUMC dataset originating from Kansas University Medical Center provides a sheer volume of high-quality images. It is essential in the field of deep learning as the model requires a huge amount of data to be trained in order to achieve high sensitivity. POLAR DB dataset is particularly useful as it includes metadata that allows for a more polarized classification of the polyps.

Though these datasets are not as commonly employed as others like Kvasir-SEG and CVC-ClinicDB, they are still quite important for the analysis of the problem of colorectal polyp analysis using computer vision and machine learning approaches. They provide more diversity in the problem, and this can improve the ability of deep learning models to perform the task of image analysis.

Table 3 provide the outline of the colonoscopy dataset.

**Table 3:** Overview of colonoscopy dataset.

Database name	Publication date	Country of origin	Access type	Access link
Kvasir	2017	Norway	Publicly Downloadable	https://datasets.simula.no/kvasir/
Kvasir-SEG	2020	Norway	Publicly Downloadable	https://datasets.simula.no/kvasir-seg/
CVC-ClinicDB	2015	Spain	Registration required	https://polyp.grand-challenge.org/CVCClinicDB/
CVC-ColonDB	2012	Spain	Registration required	https://pages.cvc.uab.es/CVC-Colon/index.php/databases/
ETIS-Larib PolypDB	2014	France	Registration required	https://polyp.grand-challenge.org/ETISLarib/
BKAI-IGH Neopolyp-Small	2022	Vietnam	Publicly Downloadable	https://bkai.ai/research/bkai-igh-neopolyp-small-a-dataset-for-fine-grained-polyp-segmentation/
Hyper-Kvasir	2020	Norway	Publicly Downloadable	https://datasets.simula.no/hyper-kvasir/
CVC-EndoSceneStill	2017	Spain	Registration Required	https://pages.cvc.uab.es/CVC-Colon/index.php/databases/cvc-endoscenestill/
CVC-300	2012	Spain	Registration Required	https://pages.cvc.uab.es/CVC-Colon/index.php/databases/
CVC-T	2015	Spain	Registration Required	https://polyp.grand-challenge.org/CVCClinicDB/
PICCOLO	2020	Spain	Registration Required	https://www.biobancovasco.bioef.eus/en/Sample-and-data-e-catalog/PD177-Databases-EN.html
LDPolypVideo	2021	China	Publicly Downloadable	https://github.com/dashishi/LDPolypVideo-Benchmark
SUN	2021	Japan	Available Upon Request	http://sundatabase.org/
SUN-SEG	2022	Japan	Publicly Downloadable	https://github.com/GewelsJI/VPS/blob/main/docs/DATA_PREPARATION.md
Kvasir-Sessile	2021	Norway	Publicly Downloadable	https://datasets.simula.no/kvasir-seg/
Kvasir-Capsule	2021	Norway	Publicly Downloadable	https://datasets.simula.no/kvasir-capsule/
Gastrointestinal Lesions in Regular Colonoscopy Dataset	2016	Spain	Publicly Downloadable	https://www.depeca.uah.es/colonoscopy_dataset/
ASU-Mayo Clinic Colonoscopy Video Database	2015	USA	Available Upon Request	https://polyp.grand-challenge.org/AsuMayo/
PolypGen	2023	UK, Norway, Egypt, Italy, France, Sweden	Registration Required	https://www.synapse.org/Synapse:syn26376615/wiki/613312
EDD2020	2020	France, Italy	Registration Required	https://ieee-dataport.org/competitions/endoscopy-disease-detection-and-segmentation-edd2020
Polypset	2021	Not Specified	Publicly downloadable	https://dataverse.harvard.edu/dataset.xhtml?persistentId=doi:10.7910/DVN/FCBUOR

Table 4 explains the polyp dataset usage across reviewed literature.

**Table 4:** Polyp dataset and their usage across reviewed literature.

S. no	Dataset name	No of papers used	References
1	Kvasir	6	(Delowar et al., 2025, Hossain et al., 2023, Yasmin et al., 2023, Duc et al., 2022, Sikkandar et al., 2024, and Al Jowair et al., 2023)
2	Kvasir-SEG	26	Islam et al. (2024), de Moura Lima et al. (2023), Wen et al. (2024), Belabbes et al. (2024), Ahamed et al. (2024), Hossain et al. (2023), Wang et al. (2025), Elkarazle et al. (2023), Ji et al. (2024), ELKarazle et al. (2025), Yuan et al. (2024), Wang et al. (2024), Fitzgerald et al. (2024), Lalinia and Sahafi (2024), Song and Shin (2024), Wan et al. (2024), Saad et al. (2023), Li et al. (2025), He et al. (2025), Wang et al. (2024), Karthikha et al. (2024), Ni et al. (2026), Sikkandar et al. (2024), Wei et al. (2024), Xia et al. (2023), and Emon et al. (2025)
3	CVC-ClinicDB	26	Islam et al. (2024), de Moura Lima et al. (2023), Wen et al. (2024), Belabbes et al. (2024), Ahamed et al. (2024), Wang et al. (2025), Elkarazle et al. (2023), Ji et al. (2024), ELKarazle et al. (2025), Yuan et al. (2024), Wang et al. (2024), Fitzgerald et al. (2024), Lalinia and Sahafi (2024), Song and Shin (2024), Raju et al. (2025), Saad et al. (2023), Li et al. (2025), Duc et al. (2022), He et al. (2025), Wang et al. (2024), Karthikha et al. (2024), Ni et al. (2026), Wei et al. (2024), Xia et al. (2023), Al Jowair et al. (2023), Hsu et al. (2021), and Jha et al. (2022)
4	CVC-ColonDB	16	Islam et al. (2024), de Moura Lima et al. (2023), Wen et al. (2024), Wang et al. (2025), Elkarazle et al. (2023), ELKarazle et al. (2025), Yuan et al. (2024), Wang et al. (2024), Lalinia and Sahafi (2024), Saad et al. (2023), Duc et al. (2022), He et al. (2025), Wang et al. (2024), Wei et al. (2024), Xia et al. (2023), Al Jowair et al. (2023)
5	ETIS-Larib PolypDB	20	Islam et al. (2024), de Moura Lima et al. (2023), Wen et al. (2024), Belabbes et al. (2024), Ahamed et al. (2024), Wang et al. (2025), Elkarazle et al. (2023), Ji et al. (2024), ELKarazle et al. (2025), Yuan et al. (2024), Wang et al. (2024), Fitzgerald et al. (2024), Lalinia and Sahafi (2024), Saad et al. (2023), Duc et al. (2022), He et al. (2025), Wang et al. (2024), Wei et al. (2024), Xia et al. (2023), Al Jowair et al. (2023), and Jha et al. (2022)
6	BKAI-IGH Neopolyp-Small	5	Ahamed et al. (2024), Ji et al. (2024), ELKarazle et al. (2025), Song and Shin (2024), and Albuquerque et al. (2022)
7	Hyper-Kvasir	4	Hossain et al. (2023), Narasimha Raju et al. (2025), Saad et al. (2023), Karthikha et al. (2024)
8	CVC-EndoSceneStill	3	Wang et al. (2025), Wen et al. (2023), and Lalinia and Sahafi (2024)
9	CVC-300	6	Ji et al. (2024), ELKarazle et al. (2025), Wang et al. (2024), Song and Shin (2024), He et al. (2025), and Wei et al. (2024)
10	CVC-T	3	Yuan et al. (2024), Duc et al. (2022), and Al Jowair et al. (2023)
11	PICCOLO	3	Saad et al. (2024), Wang et al. (2024), and Younas et al. (2023)
12	LD PolypVideo	2	Wan et al. (2024) and Li et al. (2025)
13	SUN	2	Haldar et al. (2023), Krenzer et al. (2023)
14	SUN-SEG	1	Wang et al. (2024)
15	Kvasir-Sessile	2	Saad et al. (2023), Karthikha et al. (2024)
16	Kvasir-Capsule	1	Karthikha et al. (2024)
17	Gastrointestinal Lesions in Regular Colonoscopy Dataset	1	Babuc et al. (2025)
18	ASU-Mayo Clinic Colonoscopy Video Database	1	Xia et al. (2023)
19	PolypGen	1	Ahamed et al. (2024)
20	EDD2020	1	Ahamed et al. (2024)
21	Polypset	1	Wang et al. (2024)

**Figure 4:**
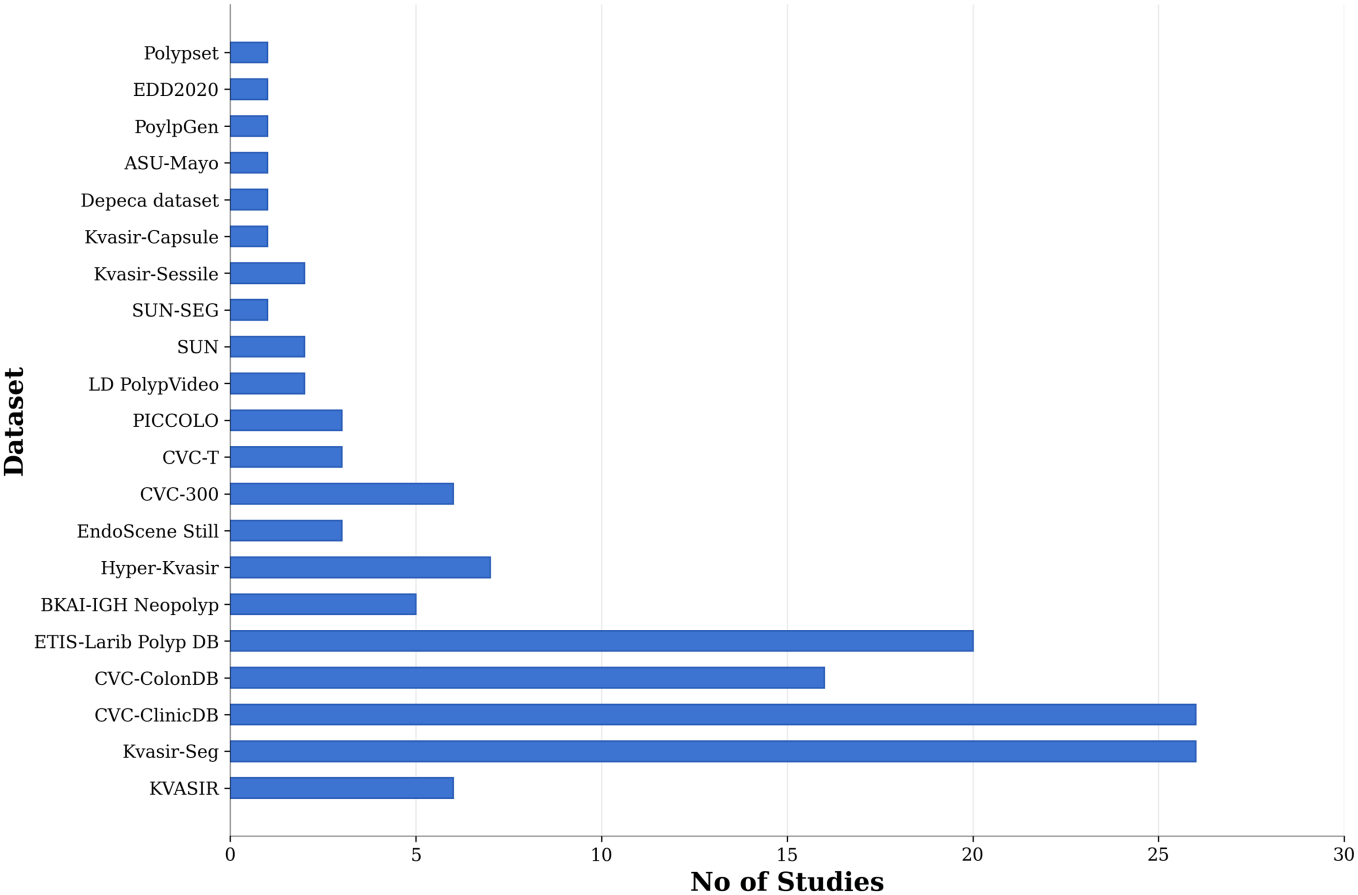
Usage frequency of publicly available polyp datasets.

Figure 4 illustrates the frequency of use of datasets in the reviewed studies. It emphasizes that Kvasir-SEG, CVC-Clinic DB, ETIS-Larib Polyp, CVC-Colon DB are the most commonly used datasets. SUN-SEG, ASU-Mayo, PICCOLO are used less due to their restricted accessibility. This visualization gives a clear impression of dataset popularity and adoption patterns in polyp detection, segmentation and classification.

## 4 Data pre-processing

Data pre-processing is an indispensable step in deep learning approaches for polyp analysis, as it has a direct impact upon feature representation, optimization, and generalization. The endoscopic images contain high variations in train and test samples, and this could significantly impact the performance of learning as it contains uncontrolled variations such as different image acquisition equipment, inconsistencies in illumination, specular reflections, motion blurs, and sensor noise (Islam et al., 2024; Ahamed et al., 2024; Hossain et al., 2023). Additionally, the unavailability of labelled samples in healthcare and high-class imbalance issues between the target and non-target areas of polyps can be risky concerning overfitting and biased learning (Yasmin et al., 2023; Younas et al., 2023).

From a methodological standpoint, pre-processing techniques can be conceived as tools that attempt to modulate and match the statistical characteristics of the input data with the inductive biasses of CNN and transformer models. Image normalization techniques can be used for improving the gradient flow and minimizing domain shift between datasets (Delowar et al., 2025; Elkarazle et al., 2023; Babuc et al., 2025). Contrast enhancement and removal, and other techniques that attempt to remove artifacts, can be viewed as attempting to raise the signal-to-noise ratio and draw attention to the minute morphological details that play a pivotal role in the definition of boundaries of a polyp (Ahamed et al., 2024; Elkarazle et al., 2023; Raju et al., 2025).

Data augmentation is a type of implicit regularization that helps a machine learn invariant feature representations against real-world variations of scale, viewpoint, lighting, and artifacts (Huang et al., 2023; Fitzgerald et al., 2024; He et al., 2025).

Notably, preprocessing in the field of polyp analysis can be non-uniform in nature and can be designed as per the specification of the task under consideration as well as the network architecture. Region-based processing like ROI extraction, creation of input, and mapping of features can be commonly utilized in the model as it can limit the model’s focus on the relevant area of differentiation (Wen et al., 2023; Haldar et al., 2023). In addition to the above, advanced techniques like mosaic data augmentation, multi-scale learning, as well as adaptive scaling, can be employed with the aim of scaling robustness in the difference in appearance of various sizes of polyps (Wan et al., 2024; Wang et al., 2024). At the same time, annotation refinement as well as data curation in the form of polygon annotation and anchor box optimization can be important in terms of enhanced annotation accuracy (Li et al., 2025).

In order to integrate all these disparate techniques, in Table 5, there is presented a structured recap of key data pre-processing approaches documented in the literature. As opposed to documenting specifics on implementations, there has been emphasis on popular patterns in methodologies and designs related to data pre-processing, where it has been treated as an enabler in undertaking reliable learning in polyp analysis systems.

**Table 5:** Key data pre-processing approaches documented in the literature.

Pre-processing category	Techniques employed	References
Image standardization	Image resizing, scaling, input size optimization, aspect ratio preservation, format standardization, grayscale conversion	Delowar et al. (2025), Islam et al. (2024), de Moura Lima et al. (2023), Wen et al. (2024), Huang et al. (2023), Chen and Ahmad (2023), Hossain et al. (2023), Elkarazle et al. (2023), Ji et al. (2024), ELKarazle et al. (2025), Wen et al. (2023), Saad et al. (2024), Wang et al. (2024), Narasimha Raju et al. (2025), Saad et al. (2023), Li et al. (2025), Duc et al. (2022), He et al. (2025), Wang et al. (2024), Ni et al. (2026), Sikkandar et al. (2024), Xia et al. (2023), Al Jowair et al. (2023), and Hsu et al. (2021)
Geometric data augmentation	Rotation, horizontal/vertical flipping, scaling, cropping, translation, shearing, affine transformation, elastic deformation, dilation, erosion	Delowar et al. (2025), de Moura Lima et al. (2023), Wen et al. (2024), Belabbes et al. (2024), Huang et al. (2023), Chen and Ahmad (2023), Wang et al. (2025), Ji et al. (2024), Fitzgerald et al. (2024), Wen et al. (2023), Wang et al. (2024), Babuc et al. (2025), Lalinia and Sahafi (2024), Narasimha Raju et al. (2025), Song and Shin (2024), Raju et al. (2025), Saad et al. (2023), Li et al. (2025), He et al. (2025), Ni et al. (2026), Younas et al. (2023), Wei et al. (2024), Xia et al. (2023), Al Jowair et al. (2023), Krenzer et al. (2023), Emon et al. (2025), and Bora et al. (2021)
Photometric augmentation	Brightness/contrast adjustment, colour jitter, hue and saturation modification, gamma correction, RGB shift	Ahamed et al. (2024), Huang et al. (2023), Chen and Ahmad (2023), ELKarazle et al. (2025), Fitzgerald et al. (2024), Babuc et al. (2025), Lalinia and Sahafi (2024), Narasimha Raju et al. (2025), Raju et al. (2025), He et al. (2025), Younas et al. (2023), Al Jowair et al. (2023), Krenzer et al. (2023), and Emon et al. (2025)
Noise injection and blur augmentation	Gaussian noise, Gaussian blur, median blur, motion blur, coarse dropout	Ahamed et al. (2024), Huang et al. (2023), ELKarazle et al. (2025), Babuc et al. (2025), Narasimha Raju et al. (2025), Raju et al. (2025), Al Jowair et al. (2023), and Krenzer et al. (2023)
Image enhancement	CLAHE, histogram equalization, image sharpening, colour correction, colour space conversion RGB↔CIELAB / YCbCr	Islam et al. (2024), Ahamed et al. (2024), Hossain et al. (2023), Elkarazle et al. (2023), Raju et al. (2025), and Bora et al. (2021)
Artifact removal	Specular reflection removal, text overlay removal using OCR + inpainting	Islam et al. (2024)
Roi-based processing	ROI isolation, centre-square transformation, bounding-box patch extraction, ROI resizing, localized depth maps	Belabbes et al. (2024), Hossain et al. (2023), Wen et al. (2023), and Haldar et al. (2023)
Advanced augmentation	Mosaic data augmentation, multi-scale training	Yasmin et al. (2023), Wang et al. (2024), Wan et al. (2024), Li et al. (2025), and Wang et al. (2024)
Annotation and dataset preparation	Polygon annotation, annotation conversion, binary mask generation, adaptive anchor box optimization k-means	Yasmin et al. (2023), Albuquerque et al. (2022), Li et al. (2025), and Bora et al. (2021)
Dataset quality and balancing	Manual removal of low-quality frames, temporal down-sampling, oversampling for class imbalance	Younas et al. (2023) and Krenzer et al. (2023)

## 5 Architecture

### 5.1 Classification architecture for polyp analysis

Image classification is an integral part of colorectal polyp analysis and focuses on differentiating polyps into relevant categories like hyperplastic and adenomatous polyps or NICE classifications. Most recent research on polyp classification has applied deep learning techniques using either Convolutional Neural Network (CNNs) or combine models of CNN and either Transformers or ensemble models consisting of both CNNs and Transformers. Applying transfer learning from pre-existing models has been quite common because of the scarcity of large medical-image folders in medical research. The trend in polyp classification architecture has shifted from simple feature extraction to sophisticated pipelines that overcome the challenges of image classification in the medical imaging domain. This section discusses the architectural evolution from custom CNN-based approaches to hybrid Transformer-based models.

#### 5.1.1 Architectural innovations in CNNs

Though the use of standard backbones is the most common practice, current research has placed greater emphasis on task-specific modifications. That captures fine structural details of hyperplastic and adenomatous polyps.

Ablation and Customization: PolyNet (Delowar et al., 2025) demonstrates an empirical approach to model development. The study proposed and tested ten different models (NewNet1 to NewNet10) to identify the most effective one. Among these, NewNet9 was selected as the base model. The subsequent addition of a self-attention mechanism represents a significant shift in the model architecture. This mechanism allows the model to weigh the relative importance of specific areas within the images. This helps the model to focus on the borders of the polyps, which are characteristics of different NICE classifications.

Interpretability through Hybrid Layers: Bionnica (Babuc et al., 2025) presents a unique approach. The design incorporates a rule-based layer on top of the convolutional and pooling stacks. This approach has been proposed as a direct response to the black-box nature of the deep learning models as it embeds medical expertise into the architecture. AM-Net (Jain et al., 2025) is a Attention based Multi-scale CNN which is designed for differentiating sporadic colonic hamartoma from adenomas using Narrow Band Imaging. The architecture’s uniqueness is due to the integration od two distinct modules: Multi-Scale Residual Network which extracts local patterns at multiple levels to detect the presence of mucosal patterns. Parallel Attention Module that incorporates both spatial and channel-wise attention highlighting the important regions to provide more attention.

#### 5.1.2 Advanced training paradigms

In order to overcome the scarcity of labelled data, recent architectures have been focused on representation learning instead of a supervised learning.

##### 5.1.2.1 Structural understanding

Huang et al. (2023) study employs a ResNet18 backbone but focused on a pretext task. Contrastive Learning in the proposed model maximizes the similarity of augmented views. Similarly the Learning Object Structure (LIO), helps the model to learn the Object Extent (localization) and Spatial Context (internal morphology). This indicates that in order to perform accurate classification in colorectal analysis, it is important to understand the geometry of the polyp.

##### 5.1.2.2 Scalability through SimCLR

The proposed framework in Wang et al. (2024) utilizes SimCLR for semi-supervised learning. By testing different backbones (VGG16, MobileNet, ResNet50, Xception), this study indicates that the choice of encoder is secondary. The quality of feature vectors learned during the unlabelled pre-training phase was important.

##### 5.1.2.3 Transfer Learning

In this Chatterjee et al. (2025) polyp detection method transfer learning have been extensively used. Pre-trained neural networks ResNet-101 and EfficientNet-B2 are trained using colonoscopy images. The use of deep hierarchical feature extraction along with compound scaling makes it possible to distinguish features. That makes it easy to differentiate hamartomatous polyps from adenomatous polyps.

#### 5.1.3 Hybrid and transformer-ensemble models

The most important recent shift is the utilization of Vision Transformers to incorporate global dependencies. That are often neglected by CNNs with their restricted receptive fields.

Contextual Relationships: CADxPloyDetect (Narasimha Raju et al., 2025) utilizes a hybrid approach. It incorporates CNNs for local feature extraction and a Transformer network to evaluate contextual relationships between image features. This approach calculates distant pixel group relationships that provides a holistic view of the lesions. In addition, the utilization of DCGAN and SMOTE helps to overcome class imbalance issues in polyp datasets.

Multi-Modal Ensembles: In the approach presented in Raju et al. (2025), a three-stage pipeline is proposed to classify images. It uses a combination of AlexNet and DenseNet-201 networks to perform feature extraction. A transformer network with an attention mechanism to refine the features. SVM is utilized to classify the features, indicates utilizing “robust” classifiers to handle high dimensional features obtained from deep networks.

#### 5.1.4 Meta learning and ensemble optimization

Rather than developing new kernels, some researchers focus on strategic usage of existing high-performance architectures.

##### 5.1.4.1 Weighted decision making

In Younas et al. (2023), the performance of six architectures was evaluated. The results showed that the performance of the weighted average between the GoogLeNet and ResNet-50 was superior to the performance of the individual architectures. This indicates that different architectures are learning different features. GoogLeNet architecture learns multi-scale features through the Inception modules. While the ResNet-50 architecture learns deep feature propagation through the skip connections.

##### 5.1.4.2 Binary decomposition

Haldar et al. (2023) simplifies the problem of multi-class polyp sizing through the usage of a One-vs-Rest approach. By combining a Binary CNN with an XGBoost classifier, the study demonstrates breaking a multi-class problem to binary decisions can improve accuracy.

Table 6 gives the summary of the classification papers about the backbone, model and special techniques used.

**Table 6:** Summary of colon polyp classification works.

Reference	Year	Backbone/model	Special techniques	Classifier task
Delowar et al. (2025)	2025	PolyNet (NewNet9)	Self-Attention, Custom CNN	Polyps’ vs. non-polyp
Huang et al. (2023)	2023	ResNet-18	Two-stage CAD, Contrastive Learning (SimCLR), LIO	Hyperplastic vs. Adenoma
Wang et al. (2024)	2024	VGG16, MobileNet, ResNet50, Xception	Semi-supervised, SimCLR, Fine-tuning	NICE classification
Babuc et al. (2025)	2025	Bionnica(CNN + Rule-based)	Rule-based layer, Patient-level thresholding	Premalignant vs. non-premalignant
Narasimha Raju et al. (2025)	2025	CNN + Transformer+SVM	Dual augmentation (DCGAN, SMOTE), Grad-CAM	Multi-class lesion classification
Raju et al. (2025)	2025	AD-22(AlexNet+DenseNet-201) + Transformer+SVM	Hybrid supervised+ unsupervised, Attention, Bounding box	Polyp classification
Haldar et al. (2023)	2023	Binary CNN + XGBoost	One-vs-Rest classification, Feature-based XGBoost	Multi-class polyp size
Younas et al. (2023)	2023	Ensemble(GoogleNet, ResNet-50, Inception-v3, Xception, DenseNet-201, SqueezeNet)	Weighted average ensemble, transfer learning	Polyp classification
Jain et al. (2025)	2025	Attention Based Multi-Scale CNN (AM-Net)	Multi-scale residual Network, parallel attention module	Differentiating sporadic colonic hamartoma from adenomas
Chatterjee et al. (2025)	2025	Transfer Learning (ResNet101 and Efficientnet B2)	Automatic sporadic colonic hamartoma characterization	Binary classification (hamartomatous vs. adenomatous)

### 5.2 Segmentation architecture for polyp analysis

Polyp segmentation is considered to be one of the most detailed level of analysis in colorectal imaging. The task demands pixel-level accuracy in defining lesion boundaries. Segmentation is different from detection or classification as it must recognize the exact shape of a lesion in an endoscopic image. One of the difficulties in analysing images in this area is the camouflage effect. The texture of lesions is found to be similar to that of healthy tissue. This similarity in texture causes difficulty in boundary detection by automated systems. Segmentation systems, in particular, find it challenging to distinguish lesions from normal tissue. To circumvent these challenges, recent studies in this area focus on developing specialized architectures. The architectures include boundary-aware decoders, hybrid transformer encoders, and foundation model-based approaches. The architectures aim at achieving better segmentation accuracy by capturing local details as well as contextual information.

#### 5.2.1 Boundary-aware and edge-guided refinement

An accurate modelling of the transition from the lesion to the surrounding tissue is vital in segmentation. Failure to effectively represent this boundary could result in boundary leakage errors by an automated system. In CRNet (Wen et al., 2024), the network is built with a Res2Net-50 backbone network as the primary feature extractor. The network is then extended with Cascaded Feature Fusion to bridge the semantic gap between the encoder and decoder features. The network is further extended with Contextual Edge Guidance. The guidance is used to generate image-level representations of the edges in the image. The edge information is used to preserve detailed structural information in the final segmentation output. In SCABNet (ELKarazle et al., 2025), the network is extended with the Spatial Gradient Boundary Attention Block. The block is used to effectively capture the spatial gradient information highlighting the transition from the lesion to the surrounding tissue. In Yuan et al. (2024), the study proposes use of a Multilevel Lesion Correction Module. The network is built with a perception and correction framework. The perception stage is used to detect errors in the segmentation output. The correction stage is used to correct the segmentation output iteratively.

##### 5.2.1.1 Reverse attention mechanisms

Reverse attention has been identified as a powerful approach for the detection of camouflaged polyps (Wang et al., 2024). The main idea behind reverse attention is the suppression of the confidently predicted area. This will ensure that the model to focus only on the undetected area. Residual Axial Reverse Attention is used in ColonFormer (Duc et al., 2022). BA-Net (Xia et al., 2023) uses the Global Reverse Attention (GRA) module for the refinement of the lesions’ boundaries. The reverse attention mechanism is further enhanced in Wang et al. (2024). The model uses the combination of Axial Patch Attention and Polyp-aware Cross-Attention in a hierarchical multi-scale reverse attention mechanism.

##### 5.2.1.2 Boundary perception modules

A number of models have focused specifically on the perception of the boundaries in the image. MPGF-Net (Ni et al., 2026) has proposed the Multi-scale Boundary Perception module. The module captures lesion’s edges at the multi-feature scale. MSNet (He et al., 2025) has proposed the Advanced Dual Feature Pyramid Decoder. The decoder is used for the integration of the multi-scale features from the various levels of the model. These models are useful for the detection of the polyp’s boundaries when they are not clear in the high-definition endoscopic image.

#### 5.2.2 Hybrid transformers and multi-scale feature fusion

Traditional convolutional neural networks perform well in learning local spatial information. However, learning long-range spatial dependencies is challenging. This challenge has been addressed by recent segmentation models that utilize hierarchical transformer-based encoders like PVT, MIT, and Swin.

##### 5.2.2.1 Hierarchical encoder architectures

UCFA-Net uses the Pyramid Vision Transformer (PVT) encoder (Wang et al., 2025). Additionally, the model uses a multi-scale cross-fusion structure. This structure ensures that feature information is not lost during the encoding process. It also improves feature interaction between different feature levels. MA-NET uses an alternative approach (Elkarazle et al., 2023). Instead of the traditional convolutional neural network encoder, the model uses the Mix Vision Transformer (MIT) backbone. This backbone is pre-trained on the ImageNet dataset. This ensures that the model can learn pixel classification with high precision using the transformer attention mechanism.

##### 5.2.2.2 Cross-fusion and global context modelling

In the hybrid architecture proposed in Yuan et al. (2024), Pyramid Vision Transformer version 2 is used together with Pyramid Hybrid Attention. This improves feature representation by using hierarchical attention mechanisms. In CPSNet, proposed in Wang et al. (2024) for camouflaged polyp detection, the Pyramid Vision Transformer is used together with the Deep Multi-scale Feature Fusion layer. This layer ensures that the model is able to learn both global context and local structural information.

##### 5.2.2.3 Dual branch architectures

In some architectures, a dual-branch structure is employed to ensure a balance between global and local feature extraction. The model presented in Fitzgerald et al. (2024) is divided into two branches. The first branch is based on SwinV2 U-Net, focusing on global hierarchical context extraction. The second branch is the Fully Convolutional Branch (FCB) designed to focus on local spatial features. The outputs of these two branches are combined, and then the final prediction is made.

##### 5.2.2.4 Adaptive and shape conscious learning

Polyp shapes may vary significantly across different patients. To cope with this issue, Polyp-ViT (Sikkandar et al., 2024) was proposed, focusing on Adaptive Deformable Convolutions in Vision Transformer. This mechanism is useful in dynamically changing the receptive field of the model according to the lesion shapes. A similar model was presented in BCL-Former (Wei et al., 2024). The model is based on Multiscale Localized Transformer Fusion (MTF) as well as a special loss function, referred to as TacLoss. The loss function is designed to handle class imbalance as well as false negative predictions.

#### 5.2.3 Multi-scale context and channel attention

The size and the shape of the polyps vary greatly. Some of them may be very small or flat, while others may be bigger and more protruding. Thus, the segmentation models need to be effective enough to capture the features from various receptive fields.

##### 5.2.3.1 Atrous convolutions and receptive field expansion

Atrous convolution is employed for the expansion of the receptive fields without compromising the spatial resolution of the image. The approach discussed in Wen et al. (2023) makes use of atrous convolution for the extraction of contextual features. BA-Net (Xia et al., 2023) and the approach discussed in Ji et al. (2024), which is based on the ResNet-50 model, have further extended the application of atrous convolution for the extraction of contextual features by employing the Enhanced Receptive Field (ERF) and the Multi-scale Context Aggregation (MCA) module.

##### 5.2.3.2 Foreground and channel feature enhancement

Foreground enhancement modules have been used for the enhancement of lesion-related features. In the paper (Ji et al., 2024), the proposed model consists of Multi-level Foreground Enhancement module, which enhances the representation of the foreground features at different levels of the feature map. The Feature Selection Fusion module has been presented in the paper (Yuan et al., 2024). This module mainly deals with the management of semantic and spatial information. Channel attention mechanisms have been widely used in the segmentation network. Some of the channel attention mechanisms are Squeeze and Excitation (SE) blocks and Locally Shared Feature (LSF) mechanisms (Saad et al., 2023). Such techniques help the model to prioritize informative lesion characteristics. The region-based techniques, like Mask R-CNN (Jha et al., 2022), make use of the multiple scale feature representation to deal with the variations in the appearance of polyps. Including hard negative examples in training improves context awareness. This enables the algorithm to discriminate between real polyps and other regions with similar appearance, thus minimizing errors.

#### 5.2.4 Foundation models and generative data strategies

##### 5.2.4.1 SAM-based segmentation refinement

A two-stage framework is presented in Islam et al. (2024) for the refinement of the medical image segmentation results. The first stage involves the application of an attention-based U-Net for the initial localization of the polyp. The network generates an initial segmentation mask. In the second stage, the generated segmentation mask is refined using the Segment Anything Model (SAM). The approach utilizes the contextual understanding at the token level for the generation of high-quality binary masks.

##### 5.2.4.2 Generative data augmentation

The problem of limited training data is solved by using Generative Adversarial Networks (GANs). The Semantic Polyp GAN, as proposed in Song and Shin (2024), is one of these architectures. It is designed to work with three different regions. The regions include polyp, non-polyp tissue, and background. This structure is effective in generating synthetic data in the form of realistic high-resolution image-mask pairs. The synthetic data is useful in improving the training of deep segmentation models. PolypSegPlus (Saad et al., 2023) is one of these architectures. The framework is designed to optimize UNet++ and ResUNet++ architectures using a comprehensive grid search.

##### 5.2.4.3 Continued evolution of U-Net architectures

U-Net architectures continue to be popular in medical image segmentation despite the rise of transformers. The evolution of these architectures is expected to continue in the future. One of these evolutions is structural changes to these architectures. One of these changes is the use of six encoder-decoder blocks with dropout as a regularization mechanism (Karthikha et al., 2024).

In summary, recent studies on polyp segmentation have shown an increasing trend from traditional CNN models to hybrid models that incorporate boundary-aware decoding, transformer-based global context learning, and multi-scale feature aggregation. Although these models have shown significant improvements in segmentation accuracy, there are challenges in dealing with extremely small and flat polyps, maintaining computational efficiency in real-time systems, and dealing with different datasets from various endoscopic devices. The future directions could involve the integration of foundation models and self-supervised learning strategies and domain adaptation to further improve robustness.

Table 7 gives the summary of the model, backbone and key contributions of segmentation papers.

**Table 7:** Summary of colon polyp segmentation works.

Reference	Year	Model/backbone	Key contributions
Islam et al. (2024)	2024	U-Net + SAM	Attention U-Net, ROI-guided SAM refinement
Wen et al. (2024)	2024	CRNet-ResNet-50	Cascaded local feature fusion, contextual edge guidance, multi-scale feature optimization, contour supervision
Wang et al. (2025)	2025	UCFA-Net, PVT	Cross-fusion, multi-scale convolutional parallel feedforward transformer, progressive attentional up sampling
Elkarazle et al. (2023)	2023	MA-Net, MixViT	Position-wise attention block, multi-scale fusion attention block
Ji et al. (2024)	2024	ResNet50	SE-residual blocks, multi-scale context aggregation modules, multi-level foreground enhancement modules.
ELKarazle et al. (2025)	2025	SCAB-Net, SwiftFormer	Feature enhancement block, channel prioritization block, spatial gradient boundary attention block
Yuan et al. (2024)	2024	Multilevel Information correction Transformer, PVTv2	Boundary attention map decoder, pyramid hybrid attention module, multilevel lesion correction module, feature selection fusion module
Wang et al. (2024)	2024	Multi-Dimensional Fusion Reverse Attention Network, Res2Net	Mobile inverted bottleneck convolutions, hierarchical multi-scale cross reverse attention,
Fitzgerald et al. (2024)	2024	FCN transformer, SwinV2 U-Net, Fully Convolutional Branch	Window-based multi-head self-attention, spatial and channel squeeze and excitation
Wen et al. (2023)	2023	Fully Convolutional Network and CNN, ResNet50	Atrous convolution, region refinement network
Song and Shin (2024)	2024	Semantic PolypGAN, StyleGAN, RenderNet	Pseudo-depth map, feature map
Saad et al. (2023)	2023	PolypSegPlus, UNet, UNet++, ResUNet and ResUNet++	Locally shared features, grid search
Duc et al. (2022)	2022	Colon-Former, mix Transformer (MiT)	Pyramid pooling, residual reverse attention
He et al. (2025)	2025	MSNet- Multi-Scale Network	Multi-scale perception, improved dual feature pyramid
Wang et al. (2024)	2024	CPSNet, Pyramid Vision Transformer	Deep multi-scale feature fusion, camouflaged object detection, multi-scale feature enhancement
Karthikha et al. (2024)	2024	Dilated U-Net-Seg,	Dilated convolutions
Ni et al. (2026)	2026	MPGF-Net, ResNet-34	Multi-scale boundary perception, pyramid feature extraction, global attention fusion
Sikkandar et al. (2024)	2024	Polyp-ViT	Adaptive deformable convolutional network, conditional positional encoding, global representation refinement
Wei et al. (2024)	2024	BCL-Former (Localized Transformer fusion with balanced constraint)	Multiscale localized transformer fusion, Tversky-based appropriate constrained loss
Xia et al. (2023)	2023	BA-Net, Res2Net50	Enhanced receptive field, brightness prior fusion, global reverse attention
Jha et al. (2022)	2022	Mask R-CNN, ResNet-101	Training on selective non-polyp regions- reduced false positive rates.

### 5.3 Detection architecture for polyp analysis

Object detection in colonoscopy uses bounding boxes to localize lesions with precision. The high variability in polyp shapes, ranging from diminutive flat lesions to large pedunculated polyps, has driven an architectural change. Traditional CNNs form the basis of feature extraction. Recent advances include multi-scale feature fusion, attention-based localization, and ensemble-based meta-learning. These methods help to overcome the limitations of traditional object detection techniques.

#### 5.3.1 Multi-scale fusion and small polyp detection

In Computer-Aided Detection, the small polyp problem represents a common challenge. Tiny lesions are mostly lost in deeper convolutions layers due to down-sampling.

##### 5.3.1.1 Enhanced single shot detectors

In SPDNET (Belabbes et al., 2024) VGG-16 backbone of SSD (Single Shot Multibox Detector) is altered. Specifically, added branch-and-concatenate modules. Using the multi-scale feature fusion and squeeze-excitation mechanisms, the model aims to enhance the detection of small polyps. It recalibrates the channel-wise features to emphasize the textures of the small polyps.

##### 5.3.1.2 Contextual receptive fields

In Wan et al. (2024), the study introduces the Contextual Receptive Field Enhancement Module (CRFEM) and the High-Dimensional Feature Compensation Structure (HDFCS) to the YOLOv8 base models. These modules are crucial for the effective application of the cross-scale features. Ensures the high-resolution features of the small polyps are not missed out by the high-level semantic data used for detection.

##### 5.3.1.3 Region-based multi-scale detection

In this research paper (Mazumdar et al., 2023), the Mask Region-based Convolutional Neural Network (Mask R-CNN) is used to automatically detect very small polyps. Using the region-proposal framework, the lesions are localized using bounding boxes from high-resolution videos. The model was trained using an augmentation strategy using real-life clinical videos along with publicly available rotated and zoomed videos. It makes the model more clinically reliable. It also helps the network to learn how to handle scale and orientation variations in reality.

#### 5.3.2 The transition to transformer-based detection

CNNs are good at feature extraction at the local level. But they encounter difficulties with global spatial information. These global features are important to differentiate between polyps and other mucosal folds or reflection artifacts.

##### 5.3.2.1 Saliency guided transformers

de Moura Lima et al. (2023) proposed a two-stage model using “Visual Saliency Transformer.” It generates saliency maps using a dense prediction transformer. Identifies prominent visual areas before passing it through ResNet-50 with a Transformer encoder-decoder architecture. This allows the model to directly predict bounding boxes using global context information rather than relying on local pixel value information.

##### 5.3.2.2 Refinement using morphological operations

This multi-stage approach proposed in de Moura Lima et al. (2023) also uses morphological operations such as hole filling and thresholding to refine the saliency maps. It shows that the hybrid approach using deep learning with morphological operations is still a powerful tool in refining localization accuracy.

#### 5.3.3 Iterative improvements in YOLO architectures

YOLO architecture remains as a standard one for real-time object detection due to their efficiency.

##### 5.3.3.1 Gradient flow and decoupled heads

The use of YOLOv8 variants (N, S, M, L, X) in Lalinia and Sahafi (2024) emphasizes the significance of the C2f group, which is based on YOLOv7’s ELAN. The use of a decoupled head for the detection and classification process allows the model to optimize the loss. This prevents the loss of gradient flow in the case of a shared head for the two objectives.

##### 5.3.3.2 Attention-driven necks

The YOLOv5 variants in Wan et al. (2024) and Li et al. (2025) have focused primarily on the “neck” of the YOLOv5 architecture. It achieves multi-scale fusion by replacing the standard neck in YOLOv5 variants with a weighted Bi-directional Feature Pyramid Network (BiFPN) (Li et al., 2025) and coordinated attention (Wan et al., 2024). This helps the models to learn the best channels for polyp detection in real-time.

#### 5.3.4 Meta-learning and ensemble stacking

A new direction in the field of detection is the use of meta-models to solve the problem of conflicting predictions made by different detectors. StackBox Ensemble: The StackBox method (Albuquerque et al., 2022) is based on the use of meta-learning techniques to stack the predictions of different detectors. Unlike the standard average-based stacking method, the predictions of the bounding box coordinates are combined in the best possible way by a learned regressor. In complex endoscopic environments single mode detectors face false positive redundancies. It is reduced by using non-maximum suppression techniques.

The present trend in polyp detection models indicates an emerging direction toward context-aware localization. The early literature focused on the “Backbone” of the models (e.g., VGG, ResNet). Recent literatures are focused on the “Neck” and “Head” of the models by utilizing BiFPNs and Decoupled Heads. These guarantee the preservation of multi-scale features. The utilization of Transformers (de Moura Lima et al., 2023) and Meta-Learning (Albuquerque et al., 2022) is an emerging direction to address the “black box” problem in polyp detection models. It provides more stable bounding box predictions that are critical in reducing the clinical false positive rates.

Table 8 summarizes the backbone, model, key architectural components and detection strategy of polyp detection papers.

**Table 8:** Summary of colon polyp detection works.

Reference	Year	Backbone/model	Key architectural components	Detection strategy
de Moura Lima et al. (2023)	2023	ResNet-50	Depth estimation, visual saliency transformer, detection transformer encoder-decoder	Two-stage saliency-guided detection
Belabbes et al. (2024)	2024	SPDNet(VGG-16)	Feature fusion, pyramidal layers, squeeze and excitation attention	SSD-based single-stage detection
Lalinia and Sahafi (2024)	2024	YOLOv8	C2f module, decoupled detection head	Real-time single stage detection
Albuquerque et al. (2022)	2022	StackBox-Multiple Detectors	Meta-learning regressor, non-maximum suppression.	Ensemble-based detection
Wan et al. (2024)	2024	YOLOv5, CSPDarknet53	Coordinated attention, path aggregation network	Attention-enhanced single-stage detection
Wan et al. (2024)	2024	YOLOv8, CSPDarknet42	Contextual receptive field enhancement, refined spatial pooling fast, high-dimensional feature compensation structure	Context-aware single-stage detection
Li et al. (2025)	2025	YOLOv5s	Squeeze and excitation attention mechanism and weighted bidirectional feature pyramid network	Multi-scale feature fusion detection
Mazumdar et al. (2023)	2023	Masked region-based CNN	Region proposal network	Automated colonic polyp detection

## 6 Evaluation metrics

### 6.1 Classification metrics

In image classification, the important task is to precisely allocate each image or instance with its correct label. Classification metrics quantify how well a model differentiates classes. These metrics include not only overall correctness but also positive and negative prediction balancing. The table below gives an overview of the most frequently used measures of classification accuracy that are identified in the studies reviewed. The most basic accuracy measures are Accuracy, Sensitivity and Specificity. The accuracy of the model represents the ratio of correctly classified samples out of total samples. Sensitivity represents the fraction of true positives that are accurately identified. Specificity indicates the percentage true negatives that are accurately detected. F1-score is the harmonic mean of precision and recall. ROC AUC measures the overall discriminatory power of the model on different thresholds. Other estimates such as the Cohen’s Kappa and Matthews Correlation Coefficient (MCC) are identified in other studies to deal with class imbalance.

Table 9 enable simple comparison across various studies highlighting the metrics used in each.

**Table 9:** Evaluation metrics used in colorectal polyp classification.

Reference	Accuracy	Precision (specificity)	Recall (sensitivity)	F1 score	ROC-AUC	Cohens kappa	MCC
Delowar et al. (2025)	✓	✓	✓	✓			
Huang et al. (2023)	✓	✓	✓	✓			
Wang et al. (2024)	✓					✓	✓
Babuc et al. (2025)		✓	✓	✓	✓		
Narasimha Raju et al. (2025)	✓	✓	✓	✓			
Raju et al. (2025)	✓	✓	✓	✓	✓	✓	✓
Haldar et al. (2023)	✓			✓			
Younas et al. (2023)	✓	✓	✓	✓	✓	✓	
Jain et al. (2025)	✓	✓		✓	✓		
Chatterjee et al. (2025)	✓		✓				

Colorectal classification has advanced from being two class problems, i.e., “polyp” vs. “non-polyp,” to multi-class histopathological classification and size estimation problems. Recent researches have incorporated hybrid architectures, semi-supervised frameworks, and domain knowledge integration to improve diagnostic accuracy and clinical interpretability.

#### 6.1.1 Binary vs multi-class performance gaps

Models focusing on binary classification achieved high performance due to reduced label complexity. Study (Raju et al., 2025) using the CVC-ClinicDB, reported a testing accuracy of 99.00% and an AUC of 0.99. Classification of visually similar polyp remains challenging but AM-Net (Jain et al., 2025) achieved good performance metrics. It achieved 86.79% accuracy, 82.84% precision, 87.75% F1-score and AUC of 0.95. Similarly (Chatterjee et al., 2025), which implement transfer learning achieved 98, 99.47% sensitivity and 85.26, 82.05% accuracy on ResNet101 and EfficientNet B2. These results indicate strong discriminative capability in simplified environments. As the number of classes increases, classification accuracy drops due to higher inter-class similarity among gastrointestinal conditions. Study (Delowar et al., 2025), which classifies 8 unique endoscopic classes achieved an accuracy and F1-score of 0.86. This indicates while overall accuracy is lower than binary models, the integration of self-attention allows for stable performance across high-dimensional label spaces.

#### 6.1.2 Hybrid and semi-supervised learning

Study (Narasimha Raju et al., 2025) introduced CADxPolyDetect, a hybrid framework combining CNN, Transformer-based contextual modelling, and an SVM. It achieved a remarkable 98% Accuracy and F1-score, suggesting that integrating transformers help in evaluating the “contextual relationships” between different lesion types. Study (Wang et al., 2024) highlights the importance of semi-supervised learning models. ResNet-backboned SimCLR model was used, it achieved an accuracy of 0.908, significantly outperforming the supervised mean accuracy of 0.808. This 10-percentage point improvement suggests that the use of semi-supervised learning can be beneficial in the use of large amounts of unlabelled endoscopic images. Study (Huang et al., 2023) demonstrates the superiority of domain-specific pretraining strategies and the use of a structured learning approach over the standard approach. The two-stage CAD system achieved an Accuracy of 0.872. It represents a 6.1 percentage point improvement over the baseline Accuracy of 0.811 obtained by the same model without the pretraining phase. It can also be observed from study (Huang et al., 2023) that a model that was pre-trained with non-medical images, specifically bird images, was able to attain an Accuracy of 0.861. This is close to that of the model that was pre-trained with polyp images. This shows that feature extraction using self-supervised methods is robust, even when pre-trained on non-medical datasets.

#### 6.1.3 Specialized tasks: histological characterization and size estimation

Recent research has moved from general polyp classification to clinically specialized diagnostic tasks, including histological classification and size estimation. Study (Younas et al., 2023) utilized an ensemble model which achieved an Accuracy of 96.3% on the Gastrointestinal Lesions in Regular Colonoscopy dataset. However, when tested on the PICCOLO dataset, the accuracy dropped to 81.2%, highlighting a significant 15.1% generalization gap. This cross-dataset variability in endoscopic imaging, remains a significant barrier to reliable clinical deployment. Study (Babuc et al., 2025) incorporates a unique “Rule-based layer” to include medical expertise. The model achieved an F1-score of 86.1% and sensitivity of 93.2%. While its F1-score is lower than the data driven models like (Narasimha Raju et al., 2025), its higher Sensitivity, clinically valuable because minimizing false negatives is critical when identifying potentially malignant lesions. Expanding the scope of classification, study (Haldar et al., 2023) utilizes XGBoosted Binary CNNs for size-based classification (0–5 mm, 5-10 mm, 10-14 mm and ≥14 mm), achieving an Accuracy of 87.01%, demonstrating the feasibility of automated size-based diagnostic support.

This quantitative synthesis demonstrates that binary polyp classification is reaching high levels (Raju et al., 2025), performance in pathological characterization remains highly dependent on dataset diversity (Younas et al., 2023). New methods based on transformer architecture and semi-supervised learning have achieved improved performance in complex multi-classification problems, with a 10% improvement in accuracy compared to traditional CNN methods.

### 6.2 Segmentation metrics

In the case of image segmentation, the target is to predict the class assignment for each pixel and to do this while capturing object boundaries effectively. Metrics used to evaluate image segmentation are based upon the comparison of the predictions with ground-truth segmentation.

Table 10 lists the segmentation evaluation criteria discussed in the analysed papers. Metrics calculated on the basis of overlap between regions take precedence in most cases. These criteria include Intersection over Union (IoU) or, alternatively, the Jaccard Index, as well as the Dice Similarity Coefficient (Dice or DSC) similarity measure. Their average versions, mIoU and mDice respectively, measure averaged performance for individual classes or images.

**Table 10:** Evaluation metrics used in colorectal polyp segmentations.

Reference	IoU	Dice	mDice	mIoU	$F_{\beta }^{\omega }$	$S_{\alpha }$	$E_{\varphi }$	**m** $E_{\varphi }$	**max** $E_{\varphi }$	MAE	PA
Islam et al. (2024)			✓	✓		✓		✓	✓	✓	
Wen et al. (2024)			✓	✓	✓	✓	✓				
Wang et al. (2025)			✓	✓	✓	✓		✓	✓	✓	
Elkarazle et al. (2023)	✓	✓									
Ji et al. (2024)	✓										
ELKarazle et al. (2025)			✓	✓							✓
Yuan et al. (2024)			✓	✓	✓	✓		✓		✓	
Wang et al. (2024)			✓	✓	✓	✓	✓			✓	
Fitzgerald et al. (2024)			✓	✓							
Wen et al. (2023)	✓	✓		✓							✓
Song and Shin (2024)			✓	✓							
Saad et al. (2023)		✓									
Duc et al. (2022)			✓	✓							
He et al. (2025)			✓	✓							
Wang et al. (2024)			✓	✓	✓	✓	✓			✓	
Karthikha et al. (2024)	✓	✓									✓
Ni et al. (2026)		✓		✓							
Sikkandar et al. (2024)	✓	✓									
Wei et al. (2024)			✓	✓							
Xia et al. (2023)			✓	✓	✓	✓	✓			✓	

Boundary-aware and structure-aware measures are also very popular. F-measure (F_β_) (Islam et al., 2024) and weighted F-measure ( $F_{\beta }^{\omega }$ ) trade off precision and recall and stress hard areas to segment. S-measure ( $S_{\alpha })$ assesses structural similarity of masks by concentrating on object shape and region consistency. E-measure ( $E_{\varphi })$ , or the so-called Enhanced Alignment Measure, takes into account both local correspondences of pixels and global image-level statistic distances. Certain works attempt to summarize E-measure values by either finding its maximum (max $E_{\varphi })$ or computing its mean( $mE_{\varphi }$ ).

Pixel-level metrics are also discussed in some of the studies. Pixel Accuracy (PA) computes the percentage of pixels that have been predicted correctly (ELKarazle et al., 2025; Wen et al., 2023; Karthikha et al., 2024). Precision and Recall assess both the incorrect and missed classifications of pixels, respectively (Elkarazle et al., 2023; Ji et al., 2024; Saad et al., 2023). Some papers also use mean precision and mean recall for evaluation (Fitzgerald et al., 2024; He et al., 2025). While these metrics provide valuable information, they remain class imbalance-sensitive and usually complemented by other metrics like overlap measures (Karthikha et al., 2024; Ni et al., 2026; Sikkandar et al., 2024).

Table 10 shows the variations in the practice of evaluation in the different segmentation studies. It provides a clear way of comparing the various metrics that are commonly used in the evaluation of segmentation in various experimental settings.

Polyp segmentation has evolved from boundary detection to handling clinical problems of small, sessile, and camouflaged polyps that are difficult to differentiate from healthy mucosa. This section synthesises the performance of three major architectural approaches: Foundation model hybrids, Transformer encoders, and improved Convolutional Neural Networks (CNNs).

#### 6.2.1 Foundation models and multi-stage refinement

An important trend is the adoption of large-scale foundation models that offer pre-trained visual priors that have been shown to be effective in cross-domain performance. The SAMU-Net model (Islam et al., 2024) leverages a customized Attention-based UNet model that incorporates a modified version of the Segment Anything Model. This model utilizes pre-trained visual priors to stabilize mask prediction. It has reported a mDice score of 0.946 and a weighted F-measure score of 0.962 on the Kvasir-SEG dataset. On the ETIS-LaribPolyp dataset, the model reported a mDice score of 0.815. This demonstrates that the foundation-model integration provides a higher performance baseline in cross data generalization. Another major approach to sharpen the lesion boundaries is iterative refinement of the mask. Iterative refinement is the main strategy used by (Wen et al., 2024; Xia et al., 2023) to further refine lesion borders beyond foundation priors. CRNet (Wen et al., 2024) achieved a mDice score of 0.912 on the Kvasir-SEG dataset with Res2Net-50 + cascaded refinement. BA-Net (Xia et al., 2023) used Brightness Prior Fusion (BF) to overcome the variance of illumination and obtained a mDice score of 0.949 along with a mean E-measure score of 0.988 on the CVC-ClinicDB dataset. Similarly, MF-RANet (Wang et al., 2024) uses multi-scale cross-reverse attention to improve the boundaries of the lesions and achieved a mDice of 0.928 on CVC-ClinicDB.

#### 6.2.2 Transformer-based global context and hierarchical modelling

Transformers are being increasingly used to overcome the receptive field limitations of conventional CNNs. It facilitates the long-range context information extraction necessary for the identification of Camouflaged Polyps. Polyp-ViT (Sikkandar et al., 2024) make use of adaptive deformable attention to dynamically change its receptive field in accordance with the morphology of the polyp, obtaining a Dice of 0.987 on Kvasir-SEG. An important transformation in this category is the Enhanced MA-Net (Elkarazle et al., 2023) which presents a modified MX-ViT transformer encoder to effectively capture ultra-fine-grained features. By incorporating a Position-wise Attention Block (PAB) and a Multi-Scale Fusion Attention Block (MFAB) in the decoder. It obtains a record mDice of 0.989 and an MAE of 0.0013 on the ETIS-Larib dataset. UCFA-Net makes use of a Pyramid Vision Transformer (PVT) along with “Cross-Fusion” modules to avoid information loss, obtaining a Dice of 0.917 on Kvasir-SEG. ColonFormer (Duc et al., 2022) makes use of a hierarchical Mix Transformer (MiT) backbone to efficiently perform multi-scale extraction, while CPSNet (Wang et al., 2024) introduce a Camouflaged Object Detection (COD) module to find hidden polyps results in a mDice of 0.829 on CVC-ColonDB.

#### 6.2.3 Evaluation via structural and object-level morphological metrics

For assessing clinical reliability beyond pixel-level overlap, recent studies focus on structural consistency, object-level alignment, and boundary-weighted precision. S-measure computes region-aware structural consistency, while E-measure computes image-level alignment. In their studies, SAMU-Net (Islam et al., 2024), UCFA-Net (Wang et al., 2025), and BA-Net (Xia et al., 2023) demonstrated high structural consistency ranging from 0.922 to 0.954 on ClinicDB. This indicates that global geometric features are well maintained by the hierarchical transformer and the foundation model decoders.

E-measure calculates image-level statistical information. A maximum E-measure of 0.988 was attained by BA-Net (Xia et al., 2023) on the CVC-ClinicDB dataset, whereas CPSNet (Wang et al., 2024) attained a maximum E-measure of 0.993. This suggests that the over-segmentation connected to conventional convolutional models have been greatly diminished by contemporary decoders.

#### 6.2.4 Hybrid strategies and data augmentation

Combining CNNs and transformers helps overcome the problem of limited annotated data and the occurrence of flat lesions. To balance the need for local as well as global features, has given rise to the development of models such as MI-Transformer and MPGF-Net. The MI-Transformer (Yuan et al., 2024), which has a mDice 0.928. The MPGF-Net (Ni et al., 2026), which uses multi-level predictive gated fusion for the reduction of redundant background noise. Thereby it obtains a high E-measure score of 0.985 on the CVC-ClinicDB dataset. Moreover, for the detection of particular clinical targets like sessile polyps, the Dilated U-Net-Seg (Karthikha et al., 2024) has obtained a high Dice score of 0.979. The PolySeg Plus (Saad et al., 2023) uses locally shared features to obtain a 0.955 Dice on CVC-ClinicDB.

### 6.3 Comparative quantitative synthesis

Transformer vs. CNN Baselines: On the Kvasir-SEG benchmark, the Transformer-based architectures, along with their hybrid variants (Elkarazle et al., 2023; ELKarazle et al., 2025; Sikkandar et al., 2024), consistently achieved a performance margin over the refined CNN baselines (Wen et al., 2024; Wang et al., 2024). While the refined CNNs stabilize at 0.91–0.93 range of mDice, the Transformer encoder, along with the use of deformable attention, has resulted in mDice >0.98. Thereby indicating an improvement of 5–7% in the accuracy of the overlapping pixels which indicates Transformers superior long-range dependency modelling.

#### 6.3.1 Generalization gap on hard datasets

The results on the ETIS-LaribPolyp dataset, which is the gold standard for small and camouflaged polyp detection, demonstrate the existence of the Generalization Gap. CNN-based models as well as their hybrid versions report scores less than 0.80 mDice. On the contrary, the foundation model hybrid SAMU-Net (Islam et al., 2024) and fine-grained transformers MA-NET (Elkarazle et al., 2023) report scores of 0.815 and 0.989, respectively. This quantitative analysis demonstrates that the hierarchical fusion of features at different scales is a necessity to overcome the low-contrast difficulties of the ETIS-LaribPolyp dataset.

#### 6.3.2 Morphological precision synthesis

The quantitative analysis of the morphological metrics shows CNNs like BA-Net (Xia et al., 2023) achieved successful balance in pixel level alignment such as E-measure 0.988. They resulted in higher MAE (0.006) compared to Transformers [MAE: 0.0013 (Elkarazle et al., 2023)]. This 78% reduction in pixel-wise error indicates the better performance of attention based decoders for surgical-grade mask precision.

### 6.4 Detection metrics

In object detection problems, in addition to determining the existence of an target, its location must be identified precisely too. The metrics used for object detection evaluations include the accuracy of classification and localization. Table 11 summarizes the results on the detection metric from the studies that were surveyed. The Average Precision (AP) is one of the standard metrics that measure precision at different levels of recall for a particular class, while Mean Average Precision (mAP) calculates their overall performance per class. mAP is presented at specific levels of IoU, like mAP@0.5 or mAP@[0.5:0.95] in some studies, while in others, a mean mAP is presented. Other standard evaluation parameters include Recall, which calculates the proportion of correctly detected instances to the total count of instances in the ground truth. Average Recall reflects the average performance of locating instances in images. F1-score is another evaluation metric combining precision and recall.

**Table 11:** Evaluation metrics colorectal polyp detection papers.

Reference	Average precision	Mean average precision	Precision	Recall	Average recall	F1score
de Moura Lima et al. (2023)	✓		✓	✓		✓
Belabbes et al. (2024)		✓	✓	✓		✓
Lalinia and Sahafi (2024)		✓	✓	✓		✓
Albuquerque et al. (2022)	✓	✓			✓	
Wan et al. (2024)			✓	✓		✓
Wan et al. (2024)		✓	✓	✓		
Li et al. (2025)		✓	✓	✓		
Mazumdar et al. (2023)		✓	✓	✓		

Table 11 shows the detection metrics used in different papers.

A direct comparison of architectural efficacy can be observed on the CVC-ClinicDB benchmark dataset. The two-stage transformer model (de Moura Lima et al., 2023) achieved an Average Precision of 0.916 and a Recall of 0.944 on the CVC-ClinicDB dataset. In contrast, the SPDNet (Belabbes et al., 2024) reported a marginally lower mAP of 0.909 and a Recall of 0.889. The ~5.5 percentage points higher Recall suggests that the transformer-based architecture may provide improved sensitivity for polyp detection in standard endoscopic frames. The YOLO-based models emphasize that internal architectural modifications often outweigh the base model version. While (Lalinia and Sahafi, 2024) uses the newer YOLO-v8m framework, it is less sensitive than the YOLOv5s + SEBiFPN setup in Li et al. (2025). The latter reached a Recall of 0.956, which is 3.9 percentage points higher than the 0.917 reported by (Lalinia and Sahafi, 2024). This demonstrates that incorporating Squeeze-and-Excitation (SE) blocks into a Bidirectional Feature Pyramid Network (Li et al., 2025) enhances attention-weighted feature fusion for detection more effectively than simply upgrading from YOLOv5 to YOLOv8.

The major issue faced by polyp detection models is shifting from static image datasets to dynamic video datasets. Study (Wan et al., 2024), which uses the CRH-YOLO model with a backbone network of CSPDarknet42, has reported a mAP score of 0.957 on the LDPolypVideo dataset. In contrast, study (Lalinia and Sahafi, 2024), which uses YOLOv8m, has reported an mAP50 score of 0.854 on a combined dataset of five benchmarks.

Though the models were trained and evaluated on different data types, the high performance reported in Wan et al. (2024) on the video dataset indicates that the model’s integration of C2F modules and the lightweight backbone (CSPDarknet42) may improve robustness to motion blur and temporal noise in the videos compared to the YOLOv8m backbone reported in Lalinia and Sahafi (2024) for static image ensemble models.

In the study (Wan et al., 2024), the authors used a customized YOLOv5 model that was tested using the local hospital dataset WCYZ. The model achieved an F1-score of 0.919. This is more quantitatively reliable than the Stacking-based Ensemble method (Albuquerque et al., 2022), which, although achieving a mAP of 0.85, suffered a considerable drop in Recall to 0.65. This highlights a key trade-off, since ensemble methods, such as Albuquerque et al. (2022), provide a solid statistical foundation but lack the high sensitivity necessary for clinical screening when compared to single-stage optimized models, such as Wan et al. (2024).

**Figure 5:**
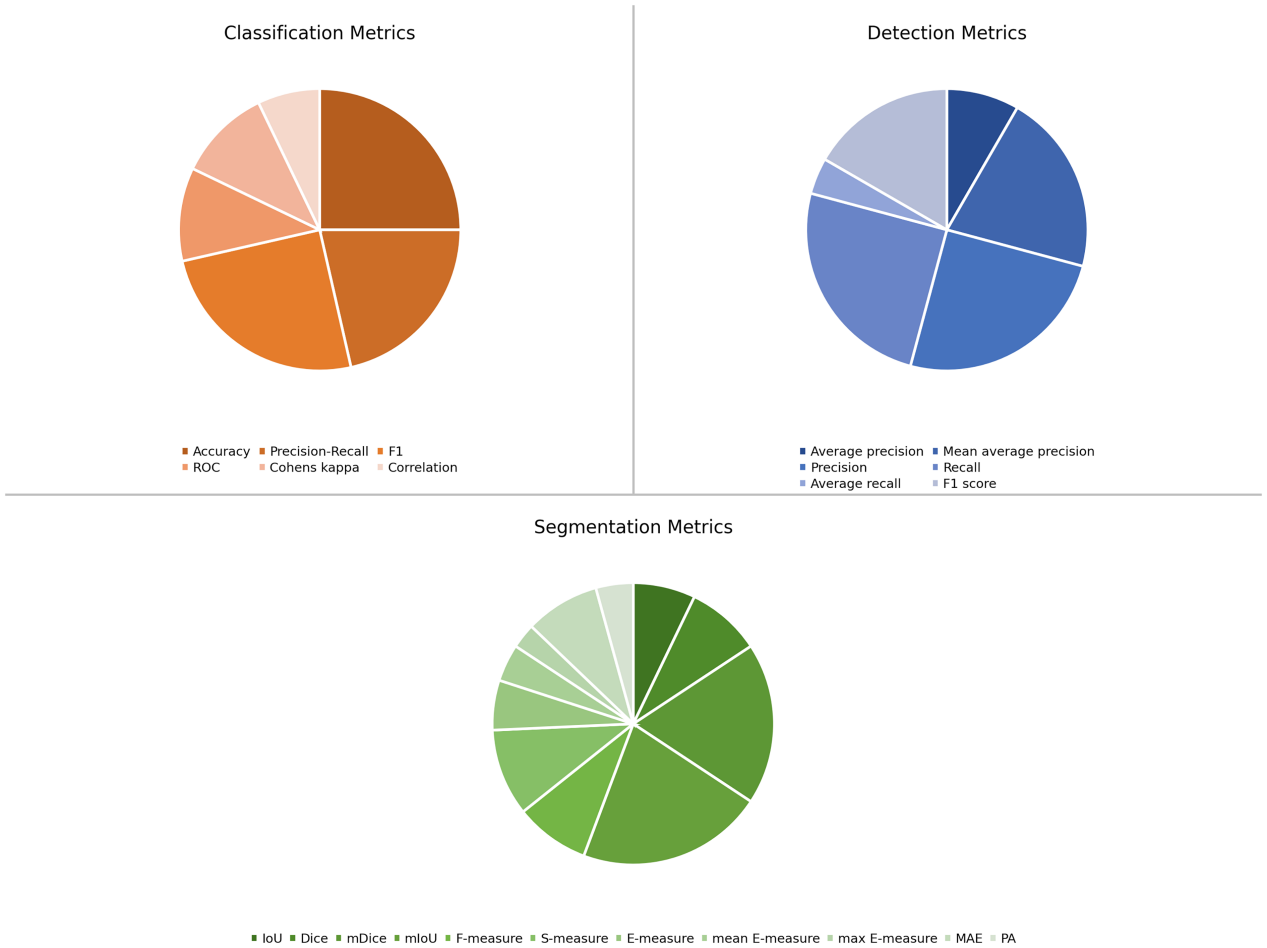
Distribution of evaluation metrics across tasks.

Figure 5 gives the distribution of evaluation metrics across the three tasks.

## 7 Strengths of the existing approaches

Colorectal AI has evolved from general computer vision to more advanced targeted systems. Modern techniques have proven their effectiveness in overcoming specific challenges in clinical practice. Challenges like missing rate of flat lesions and increased false positive rates can be handled effectively.

### 7.1 Multi-scale precision and morphological resilience

A major improvement in the contemporary detection and segmentation researches is the shift toward hybridization. Global–local fusion: The combination of Transformer models and CNNs (Keum and Giovannucci, 2019; Lee et al., 2020; Sánchez-Peralta et al., 2020; Hossain et al., 2023; Fitzgerald et al., 2024) facilitates in extracting both fine local texture and the capturing long-range spatial dependencies. This is a major requirement in the segmentation of irregularly bordered or camouflaged polyps that blend with the surrounding mucosa (Ma et al., 2021; de Moura Lima et al., 2023). Resilience to Scale variation: The combination of pyramid encoders and deformable attention (An et al., 2022; Elkarazle et al., 2023) is robust against the wide variability encountered in polyps. Thus, sessile polyps, which have been hard to detect, can now be detected with the same precision as larger, pedunculated lesions (Silva et al., 2014; Ji et al., 2022).

### 7.2 Discriminative depth in classification

#### 7.2.1 Background suppression

Attention mechanisms work to remove background distractions (Mármol et al., 2017; Jha et al., 2019; Delowar et al., 2025; Wang et al., 2024). In the case of polyp patterns, they allow the model to focus on pit patterns and vascular structures. This enables better confident classification of the tissue as neoplastic or non-neoplastic. Boundary and Edge Preservation: Explicit boundary supervision and edge-guided learning resulted in a marked reduction of false positive cases on the mucosal folds (Ali et al., 2020; Ahamed et al., 2024; ELKarazle et al., 2025). Defining the polyp-mucosa interface mathematically, models avoid frequent error of misclassifying reflections of normal tissues as lesions.

### 7.3 Generalization and clinical trust

#### 7.3.1 Cross-dataset and hospital-acquired validation

Recent methodologies include generalization and applying a model to the real world (Nisha et al., 2022; Misawa et al., 2021; Chen and Ahmad, 2023). These methods helped to create models that work in varying sensor conditions, noise, and light interference when tested on various endoscopic images. Explainable AI (XAI) and Interpretability: The combination of attention map visualizations and Grad-CAM (Duc et al., 2022; Wang et al., 2024) has enabled a new level of clinical interpretability. Instead of being a ‘black box’, clinicians are able to see the AI’s reasoning, and can therefore adopt these models to assist (Islam et al., 2024).

### 7.4 Deployment and real-time assessments

While previous models had a trade-off between prioritizing accuracy and adding latency, new models have a better efficiency across the board.

Inference Speed: The use of light backbones and partial decoders (Tharwat et al., 2022; Smedsrud et al., 2021; Wang et al., 2025) allow for true real-time performance. AI can run during the procedure at the normal endoscope frames, 30–60 FPS without disrupting flow. Methodological Rigor: The emphasis on ablation studies and testing for statistical significance (Tajbakhsh et al., 2015; Belabbes et al., 2024) means the strengths of a model are not a product of a specific dataset, but are true mathematically and clinically.

## 8 Limitations and challenges

Despite the technical milestones in colorectal AI, some systemic limitations remain. That challenge the transition from research prototypes to real-world clinical deployment. These barriers fall into three major areas: data-related bias, trade-offs related to the complexity of the architecture, and the challenges related to moving into temporal video analysis.

### 8.1 Dataset bias and generalization gap

A principal area of concern is the lack of multi-center diversity. Most of the reported state of the art performances have been observed on datasets collected from a small number of institutions (Morgan et al., 2023; Wang et al., 2020; Ali et al., 2021). This creates an inherent risk of dataset-specific bias. The model may achieve great results on one center’s endoscopic equipment, but will perform low on alternate unseen benchmarks (Pogorelov et al., 2017; Wen et al., 2024; Yuan et al., 2024). Aggressive preprocessing and exclusion of clinically challenging samples may result in overestimation of real-world clinical performance (Bernal et al., 2012; Huang et al., 2023). The performance accuracy degradation and lack of robustness is visible in adverse imaging conditions such as motion blur, specular highlights and shadows (Kather et al., 2019; Islam et al., 2024).

### 8.2 Computational complexity and deployment feasibility

The high computational and memory cost primarily applies to a limited number of complex models (Borgli et al., 2020; Ji et al., 2024). In particular transformer and multi-module hybrid architectures. While these models may achieve high levels of performance, their high parameter counts, high FLOPs, and increased inference can limit their deployment on clinical hardware systems. Some research has been directed toward lightweight architectures that facilitate real-time inference such as the MobileNet, SwiftFormer, and lightweight variants of U-Net (Tharwat et al., 2022; ELKarazle et al., 2025). The primary issue is thus not the availability of efficient models, but the lack of a unified framework for the reporting of computational metrics, and in particular, inference latency and memory usage. This makes it difficult to assess the practical deployment of various model in real-world clinical environments (Saad et al., 2024).

### 8.3 Static image-based vs. video-based analysis

A substantial part of the existing research has continued to be focused on static image-based evaluation. This does not entirely capture the dynamic nature of the real-time colonoscopy procedure (Shin et al., 2018; Ji et al., 2022). Colonoscopy is a video-based procedure. However, only a limited number of models have been assessed in terms of temporal consistency. Without utilizing temporal dependencies between consecutive video frames, computer-aided detection systems have the potential for varying detections. The polyp is identified in one video frame but goes undetected in the next due to the movement of the camera and the angle of view. One of the major challenges in the development of video-based AI systems is the scarcity of expert-annotated colonoscopy video datasets (Wen et al., 2023). Systems should be capable of performing high level consistency across a series of video frames and not individual images. That improve the potential of such systems in assisting the colonoscopy procedure.

### 8.4 Rare polyp morphology

Despite the high emphasis on easily detectable polyps in existing research, rare types of polyps and small polyps have been less studied because of poor representation in the training datasets (Ma et al., 2021). Additionally, some methods have been based on backbone networks that have been pre-trained on non-medical datasets such as ImageNet. They might lack the degree of adaptability required for detecting the nuances of texture that occur in mucosal pathology and other gastrointestinal abnormalities.

## 9 Future research directions

Future work in colon polyp segmentation should extend beyond architectural improvements. Instead, it should focus on addressing challenges in terms of efficiency, generalization, and practical problems. Although the performance of the current developments has shown good results on the available benchmarks, their robustness and applicability in different real-world clinical scenarios is an area under investigation. Although some commercial CAD systems, such as EndoBRAIN, have shown the feasibility of AI-assisted colonoscopy, some research systems need further validation.

Domain generalization and cross-institution learning are areas that require further scientific exploration. This is because colonoscopy imagery is diverse owing to the use of different endoscopes, lighting settings, the extent of bowel preparation, and individual patient anatomy. Most models developed today use single institution datasets often show limited generalization when applied under unseen conditions found in a clinical environment. Future works should consider tasks like domain adaptation learning and cross-validation on more than one dataset. Self-supervised and weakly supervised techniques would allow researchers to overcome the need for large-scale pixel-level annotations.

Another cardinal direction lies in lightweight and deployment ready architectures. Transformer-based and hybrid CNN–Transformer models have reported high accuracy in segmentation tasks, but these have usually required high computational and memory resources. This can limit their deployment in real-time clinical environments. In the future, there should be more efforts regarding parameter-efficient attention mechanisms, model compression, knowledge distillation techniques, while hardware-aware optimization can also enhance support for real-time performance. Inference speed, memory usage, and computational cost should be reported by default.

Given that colonoscopy is essentially a video-based procedure, moving beyond static image analysis is important. Most of the existing approaches evaluate the models on an individual frame-by-frame basis. This ignores the temporal dependencies across successive frames. Future work should look at temporal consistency modelling and motion-sensitive attention mechanisms. Architectures like Video Transformers and recurrent ones can help in modelling interdependencies between frames. Such models can reduce instability in prediction and transient false positives during live procedures.

The approach that utilizes foundation models and prompt-based segmentation models also holds very promising results. Large foundation models have strong generalization capabilities. If such models are tuned adequately, then there would be less need to rely on medical annotations. Future studies would focus on fine-tuning and design of prompt approaches in the context of colonoscopy data. It will be a challenge to retain the boundary definition and anatomical structure.

How these predictions can be explained and measured for uncertainties is also important for adoption in the medical field. Deep learning algorithms for segmentation should have capabilities beyond the usual positive and negative mask specifications. Such systems should also quantify prediction confidence and uncertainty. Bayesian neural networks can handle uncertainty while making predictions.

There should be better focus on validation studies and translational studies. Indeed, currently, the majority of studies involve evaluation with publicly available datasets on past studies. Future research should include prospective studies and clinician validation to better assess real-world clinical utility.

Future research may integrate imaging-based analysis with molecular-level cancer studies. Computational approaches that analyses transcriptomic and genomic interactions in cancer cells (Eralp and Sefer, 2024; Eralp and Sefer, 2025). These highlight the potential of combining biological data with medical imaging for improved understanding of colorectal cancer progression.

## 10 Conclusion

This review carried out a detailed examination of deep learning-based models for colorectal polyp classification, detection, and segmentation, including CNN, Transformer, and hybrid models. Although CNN models still prove to be reliable for local feature extraction, the Transformer and hybrid models are shown to perform better for modelling global information and dealing with complex polyp conditions. However, despite the performance improvements, issues including lack of diversity among datasets, poor generalization among different datasets, high computational complexities, and model fragmentation for specific tasks still pervade among the three tasks. The key areas that need to be addressed for advancing deep learning models for polyp analysis systems from laboratory environments to practical applications include a focus on unified multi-task models, enhanced generalization techniques via extensive and multi-institutional datasets, reduced computational complexities for real-time implementations, and meaningful performance assessments.

## Data Availability

Publicly available datasets were analyzed in this study. This data can be found at: https://datasets.simula.no/kvasir-seg/.
